# Nuclear receptor ERRα and transcription factor ERG form a reciprocal loop in the regulation of *TMPRSS2:ERG* fusion gene in prostate cancer

**DOI:** 10.1038/s41388-018-0409-7

**Published:** 2018-07-24

**Authors:** Zhenyu Xu, Yuliang Wang, Zhan Gang Xiao, Chang Zou, Xian Zhang, Zhu Wang, Dinglan Wu, Shan Yu, Franky Leung Chan

**Affiliations:** 1 Department of Pharmacy, Yijishan Affiliated Hospital, Wannan Medical College, Wuhu, Anhui China; 2 School of Biomedical Sciences, Faculty of Medicine, The Chinese University of Hong Kong, Hong Kong, China; 3 Department of Pharmacology, Southwest Medical University, Luzhou, China; 4 Clinical Medical Research Center, Shenzhen People’s Hospital, Second Clinical Medical College, Jinan University, Shenzhen, China

## Abstract

The *TMPRSS2:ERG* (T:E) fusion gene is generally believed to be mainly regulated by the activated androgen receptor (AR) signaling in androgen-dependent prostate cancer. However, its persistent expression in castration-resistant and neuroendocrine prostate cancers implies that other transcription factors might also regulate its expression. Here, we showed that up-regulation of nuclear receptor estrogen-related receptor alpha (ERRα) was closely associated with the oncogenic transcription factor ERG expression in prostate cancer, and their increased coexpression patterns were closely associated with high Gleason scores and metastasis in patients. Both ERRα and ERG exhibited a positive expression correlation in a castration-resistant prostate cancer (CRPC) xenograft model VCaP-CRPC. We showed that ERRα could directly transactivate T:E fusion gene in both AR-positive and -negative prostate cancer cells via both ERR-binding element- and AR-binding element-dependent manners. Ectopic T:E expression under ERRα regulation could promote both in vitro invasion and in vivo metastasis capacities of AR-negative prostatic cells. Intriguingly, ERG expressed by the T:E fusion could also transactivate the ERRα (*ESRRA*) gene. Hereby, ERRα and ERG can synergistically regulate each other and form a reciprocal regulatory loop to promote the advanced growth of prostate cancer. Inhibition of ERRα activity by ERRα inverse agonist could suppress T:E expression in prostate cancer cells, implicating that targeting ERRα could be a potential therapeutic strategy for treating the aggressive T:E-positive prostate cancer.

## Introduction

The breakthrough discovery of fusion genes formed by recurrent chromosomal rearrangements in prostate cancer in past decade contributes significantly to our current conceptual understanding on the significance of androgen signaling in prostate carcinogenesis [1], and also makes considerable impact on the early diagnosis, prognosis, and potential therapeutic management of this cancer [2, 3].

These recurrent fusion genes prevalent in prostate cancer are formed by fusion of the promoter and 5′-untranslated region (UTR, exon 1 or 2) of the androgen-regulated prostate-specific transmembrane protease serine 2 gene (*TMPRSS2*) with the translated regions of members of erythroblastosis virus E26 transforming sequence (ETS) family of transcription factors, *ERG* and *ETV1* [1]. The consequence of these rearrangements results in pathogenic overexpression of ETS transcription factors and activation of transcriptional programs in an androgen-dependent manner, driving the oncogenic progression of prostate cancer. Subsequently, more fusion genes are identified in prostate cancer, involving additional androgen-responsive 5′-partners and other ETS and non-ETS members [4]. Among these fusion genes, *TMPRSS2:ERG* (T:E) fusion is the most dominant rearrangement and prevalent in majority of prostate cancer patients (40–70%) [5–8]. Association studies show that T:E fusion-positive prostate cancer patients present more aggressive clinical phenotypes, in term of higher tumor grade and metastasis potential, progression to androgen-independence, shorter survival and unfavorable prognosis [5, 9–13]. Clinical and preclinical studies demonstrate that T:E fusion is detected in premalignant prostatic intraepithial neoplasia (PIN) lesions [9] and targeted in vivo expression of ERG can induce PIN lesions in transgenic mouse prostate [14], suggesting that ERG plays a casual role in prostate cancer initiation. Thus, T:E fusion or *ERG* is regarded as a key oncogene in prostate cancer [15].

Since most prostate cancer-prevalent fusion genes are formed by the fusion with the androgen-responsive promoter region of the androgen-activated genes (e.g., *TMPRSS2* and *SLC45A3*), it is thus generally regarded that these fusion genes are mainly regulated by androgen receptor (AR) [1] and their oncogenic activation or overexpression is attributed to the activation of AR signaling in androgen-dependent prostate cancer. However, these fusion genes or ETS members are also expressed in androgen-independent and AR-negative prostate cancer xenograft tumors [16] and cell lines (e.g., NCI-H660) [17], metastatic androgen-independent prostate cancer [12], castration-relapse prostate cancer [18], neuroendocrine prostate carcinoma [19, 20], and also patient-derived prostate cancer stem cells [21]. It still remains puzzling how these androgen-responsive fusion genes are transcriptionally regulated in androgen-independent or AR-negative prostate cancer cells. These puzzled observations also implicate that these fusion genes may bypass the AR regulation in advanced prostate cancer, and also these fusion genes-positive patients are susceptible to evolve into therapy-resistant and androgen-independent metastatic disease.

Previously, a genome-wide gene expression study on T:E fusion-positive prostate cancer samples performed in two prostate cancer cohorts identifies a specific T:E fusion gene signature that is associated with estrogen receptor (ER) signaling and also the T:E fusion expression could be modulated by ER ligands in AR-negative NCI-H660 cells, implying that the T:E expression can be regulated by an ER-dependent mechanism [22]. In addition, an in vitro study shows that the T:E expression in AR-positive VCaP cells can be induced by vitamin D receptor ligands [23]. Based on these limited indirect studies, it suggests that besides AR, other nuclear receptors or transcription factors could also regulate the T:E fusion gene in prostate cancer.

Estrogen-related receptor alpha (ERRα, NR3B1, and ESRRA) is a ligand-independent orphan member of the nuclear receptor superfamily. Studies in past decades indicate that together with its coactivator PGC-1α, ERRα functions as a key transcriptional regulator of energy homeostasis and mitochondrial functions [24]. ERRα displays an increased expression pattern in hyperplastic prostates and advanced prostate cancer [25], and is also implicated as a negative prognostic predictor for prostate cancer [26]. Our recent study shows that ERRα promotes the hypoxic growth adaptation of prostate cancer cells via a mechanism of direct interaction with HIF-1α and augmentation of HIF-1 signaling [27]. An in vivo study on intraosseous prostate cancer xenograft model shows that ERRα promotes the metastatic growth of prostate cancer lesions in bone via its modulation of stromal microenvironment [28].

In this study, we uncovered a reciprocal regulatory loop between ERRα and ERG that can promote synergistically the advanced progression of prostate cancer in an AR-independent manner, and suppression of T:E/ETS fusion genes via ERRα targeting could be a potential therapeutic strategy for prostate cancer.

## Results

### Both ERRα and ERG show up-regulation and also a positive correlation in advanced prostate cancer

**Fig. 1:**
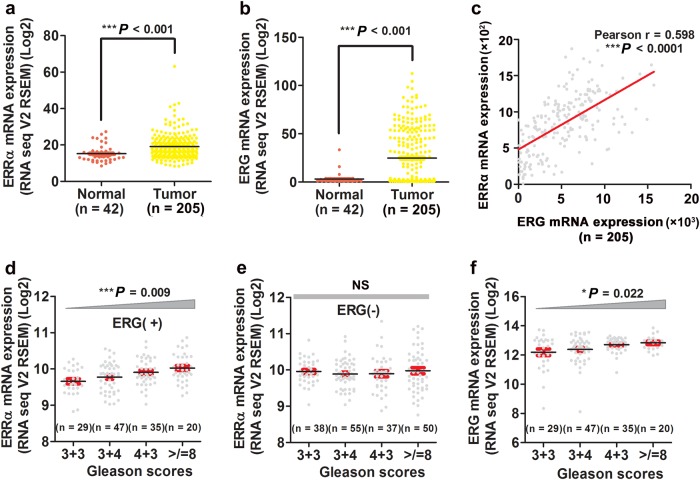
Increased expressions of ERRα and ERG are correlated with advanced stage of prostate cancer. (**a** and **b**) Expression profiles of ERRα and ERG as revealed from RNA-seq datasets from cohorts of prostate cancer patients. Both ERRα and ERG exhibited significant higher expression patterns in prostate tumor tissues (*n* = 205) than normal prostatic tissues (*n* = 42), as revealed from RNA-seq datasets from the Cancer Genome Atlas. The genomic profile information on the samples is available from the cBioPortal website (http://www.cbioportal.org/). ^***^P < 0.001 versus normal prostate. (**c**) Pearson correlation and linear regression analysis between ERRα and ERG expressions in primary prostate cancer samples (*n* = 205). Results showed that mRNA levels of ERRα and ERG displayed a positive correlation in prostate cancer samples. Relative Pearson correlation coefficient *r* = 0.598, *P* < 0.0001. (**d** and **e**) mRNA expressions of ERRα exhibited statistically significant positive correlation with the Gleason scores in ERG-positive but not in ERG-negative prostate cancer patients. NS nonsignificant. (**f**) mRNA expression of ERG exhibited statistically significant positive correlation with Gleason scores in prostate tumor tissues

In order to elucidate whether increased expressions of ERRα and ERG expressed by the T:E fusion gene would play a role in the advanced progression of prostate cancer, we analyzed the expression profiles of ERRα and ERG in clinical prostate tumor tissues and their association with the tumor grade and metastasis of patients using the cancer genomic datasets from the Cancer Genome Atlas. Analysis of RNA-seq data from 205 prostate cancer patients and 42 normal male individuals totally showed that the mRNA levels of both ERRα and ERG displayed significant higher expressions in tumor tissues as compared to that in normal prostatic tissues (Fig. 1a, b). Analysis also showed that both ERRα and ERG manifested a positive expression correlation in prostate cancer (Fig. 1c). Further analysis of the association between ERRα and ERG expressions in AR^low^ and AR^high^ subsets of prostate cancer patients in the same datasets revealed that both ERRα and ERG exhibited a positive correlation in both subsets of patients (Supplementary Fig. S1a, b). Expression analysis showed that increased ERRα expressions exhibited a significant positive correlation with the Gleason scores in ERG-positive but not ERG-negative prostate cancer patients and higher ERRα was also shown in metastatic prostate cancer tissues (Fig. 1d, e; Supplementary Fig. S1c), and associated with shorter survival of prostate cancer patients [26]. Similarly, a statistically significant positive correlation was demonstrated between elevated ERG expression and the Gleason scores in cancer patients (Fig. 1f). Together, these results showed that up-regulation of both ERRα and ERG was positively correlated with the Gleason scores and metastasis in prostate cancer tissues.

### Castration-relapse xenograft tumors exhibits increased expressions of ERRα and ERG

**Fig. 2:**
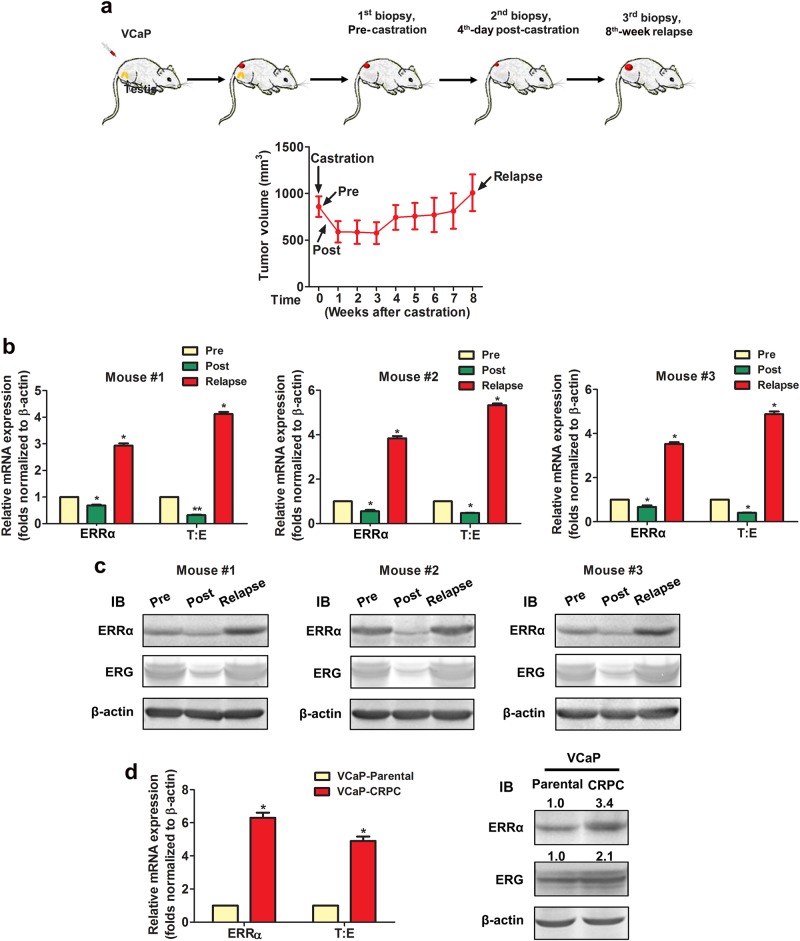
Both ERRα and ERG exhibit a positive increased expression correlation in VCaP-CRPC castration-resistant xenograft tumors. **a** VCaP-CRPC xenograft model. Upper panel: schematic diagram shows the growth progression of VCaP xenograft tumors in castrated host mice and time-points of tumor biopsies performed. Lower panel: growth curve of VCaP xenograft tumors in intact host mice (with tumor sizes of 1 cm^3^ at precastration; Pre), fourth day postcastration (Post) and relapse at eighth week postcastration (Relapse) (*n* = 5). **b** RT-qPCR analysis of ERRα and T:E fusion gene and (**c**) immunoblots of ERRα and ERG expressed in VCaP-CRPC xenograft tumors. mRNA and protein levels of both ERRα and ERG expressed by T:E fusion dropped significantly at fourth day postcastration and rebounded significantly in relapsed tumors. **d** RT-qPCR and immunoblot analyses of ERRα and T:E (ERG) in VCaP-CRPC xenografts-derived cell subline. Both ERRα and T:E/ERG expressed at significant higher levels in VCaP-CRPC cells as compared to their parental VCaP cells. ^*^P < 0.01 versus precastration VCaP tumors or parental VCaP cells. All data are presented as mean ± SEM and obtained from three independent experiments

In order to elucidate the expression correlation between ERRα and ERG in the progression of castration-resistant prostate cancer (CRPC), we established a CRPC xenograft tumor model VCaP-CRPC based on the castration-relapse growth of AR- and T:E-positive VCaP cells (Fig. 2a). Analysis of ERRα and ERG expressions in VCaP-CRPC tumor biopsies revealed that mRNA and proteins levels of both ERRα and ERG declined at fourth day postcastration but their levels rebounded significantly in castration-relapse xenograft tumors at levels higher than precastration (Fig. 2b, c). We also established an androgen deprivation-resistant VCaP-CRPC cell line derived from the VCaP-CRPC xenograft tumors. Expression analyses showed that similar to VCaP-CRPC tumors, VCaP-CRCP cells exhibited significant higher endogenous expression levels of ERRα and ERG as compared to their parental VCaP cells (Fig. 2d). These results suggest that both ERRα and ERG exhibit an apparent positive expression correlation in CRPC xenograft tumors and their enhanced coexpressions may play a role in the CRPC.

### T:E fusion gene is positively regulated by ERRα in prostate cancer cells independent of their AR expression status

**Fig. 3:**
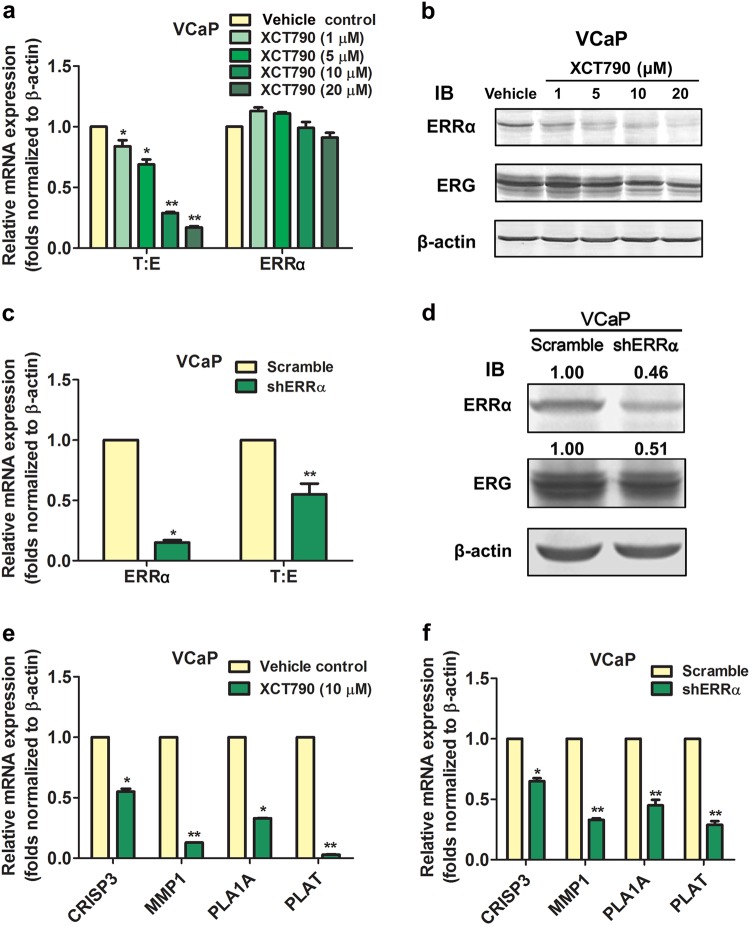
Suppression of ERRα reduces T:E fusion or ERG expression and ERG-mediated signaling in AR-positive VCaP cells. (**a, c**) mRNA and (**b, d**) protein expressions of T:E fusion/ERG in VCaP cells upon XCT790 treatment or ERRα knockdown by shRNA. Both mRNA and protein levels of T:E fusion/ERG were significantly reduced by XCT790 treatment in a dose-dependent manner (1–20 µM) and also shERRα. (**e, f**) mRNA levels of ERG-responsive targets were significantly suppressed by either XCT790 or shERRα. ^*^P < 0.05; ^**^P < 0.01 versus vehicle treatment or Scramble shRNA transfection

**Fig. 4:**
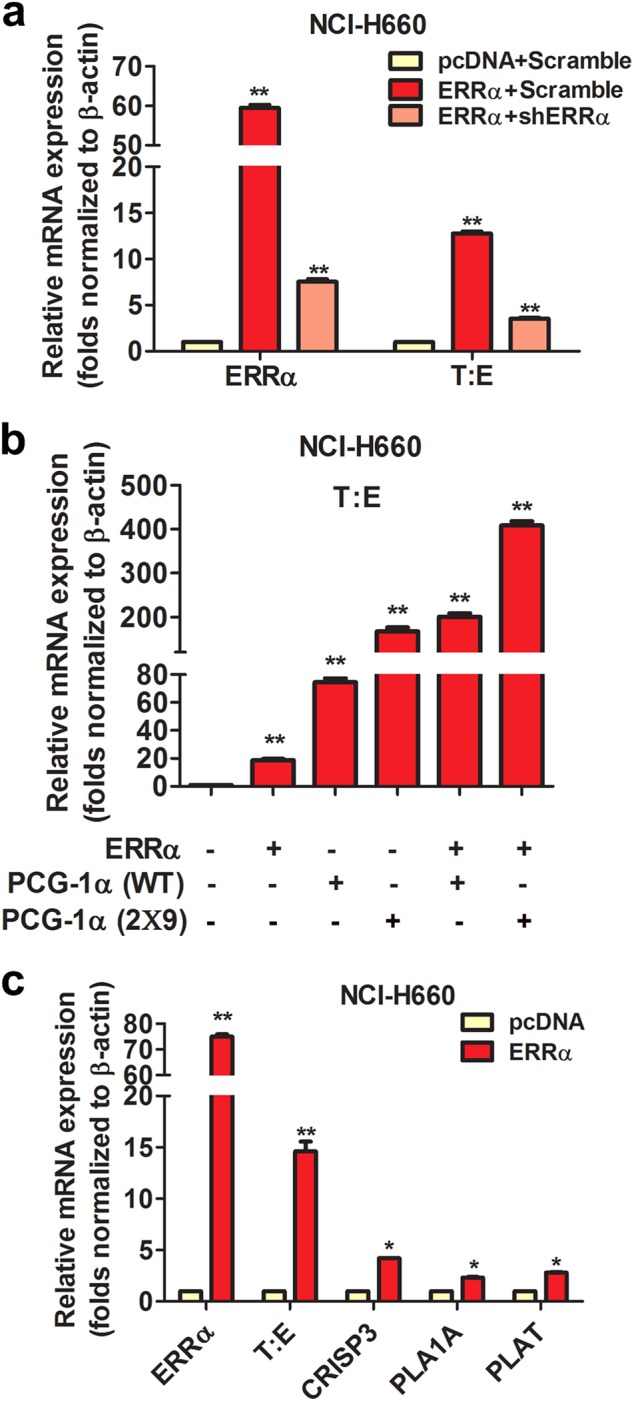
ERRα overexpression increases T:E fusion or ERG expression and enhances ERG-mediated signaling in AR-negative NCI-H660 cells. **a** mRNA levels of T:E fusion could be significantly increased by transient ERRα transfection but attenuated by cotransfection with ERRα and shERRα. **b** ERRα-induced increase of T:E fusion mRNA levels could be further enhanced by co-transfection with coregulator PGC-1α or its ERRα-specific mutant PGC-1α (2 × 9). **c** mRNA levels of ERG-responsive targets were significantly elevated by ERRα transfection. ^*^P < 0.05; ^**^P < 0.01 versus empty vector pcDNA3.1

In order to demonstrate whether the T:E fusion could be regulated by ERRα, we examined the effects of suppression of ERRα activity or its overexpression in two prostate cancer cell lines, VCaP and NCI-H660, both harbor the T:E fusion gene and express T:E transcripts and ERG protein [1, 29]. In vitro treatment with an ERRα-selective inverse agonist XCT790 showed that XCT790 could dose-dependently suppress both the mRNA and protein levels of fusion ERG in AR-positive VCaP cells (Fig. 3a, b). Treatment with low-dose XCT790 (1 µM) could also decrease the mRNA and protein levels of fusion ERG in VCaP cells in a time-dependent manner (Supplementary Fig. S2a, b). However, the XCT790-induced protein reduction of ERRα in VCaP cells could be prevented by a proteasome inhibitor MG132 (Supplementary Fig. S2c), suggesting that such ERRα reduction was mediated by an enhanced proteasomal degradation mechanism as similarly reported in MCF7 breast cancer cells [30]. Similarly, transient ERRα knockdown could significantly decrease both the mRNA and protein levels of fusion ERG in VCaP-shERRα transduced cells (Fig. 3c, d). Suppression of ERRα by either XCT790 or shERRα could significantly diminish the mRNA levels of multiple ERG-responsive target genes, including *CRISP3, MMP1*, *PLA1A*, and *PLAT* (Fig. 3e, f), suggesting that ERG-mediated signaling was restrained by ERRα suppression. ERRα knockdown also suppressed the expressions of ERRα targets in both VCaP and LNCaP cells (Supplementary Fig. S3a, b). Since another ERR isoform ERRγ shares certain overlapping functions with ERRα in metabolic reprogramming in cancer cells [31], we then examined whether ERRγ knockdown could affect the T:E fusion expression in prostate cancer cells. Results showed that transient ERRγ knockdown induced no change in T:E fusion expression in VCaP cells (Supplementary Fig. S3c). No detectable change in mRNA levels of ERG-responsive targets was shown in T:E-negative LNCaP cells and also no change in AR levels in VCaP cells upon XCT790 treatment or ERRα knockdown (Supplementary Fig. S4). Since T:E fusion gene possesses the same promoter of *TMPRSS2* gene, we also determined that suppression of ERRα activity by XCT790 could decrease the endogenous *TMPRSS2* transcripts in both T:E-positive VCaP and T:E-negative LNCaP cells (Supplementary Fig. S2d, e). In order to provide an insight into whether AR signaling would be involved in the ERRα-mediated regulation of T:E expression, we next examined the effects of inhibition of AR activity (by flutamide or enzalutamide) or AR knockdown (siAR) and XCT790 treatment in T:E suppression in VCaP cells. Our results showed that XCT790-induced T:E suppression at similar or lower magnitude as AR antagonist or siAR in VCaP cells, with or without stimulation with AR agonist (R1881 or DHT), and with further T:E suppression by combined XCT790-AR antagonist/siAR treatments (Supplementary Fig. S5). These results suggest that suppression of T:E expression by ERRα inhibition in AR-positive prostate cancer cells is independent of AR or the involvement of AR is minimal in this suppression. To further elucidate the significance of ERRα on the regulation of T:E fusion in AR-negative prostate cancer cells, we then examined the effect of ectopic overexpression of ERRα in NCI-H660 cells. Results showed that transient transfection of ERRα could significantly increase the mRNA levels of T:E fusion transcripts, with further enhancement by cotransfection with either its wild-type coregulator PGC-1α or ERRα-specific mutant PGC-1α(2 × 9) but attenuated by ERRα knockdown, in NCI-H660 cells (Fig. 4a, b). Overexpression of ERRα could elevate the mRNA levels of multiple ERG-responsive targets (Fig. 4c). Together, these results suggest that T:E fusion or ERG expression and its downstream ERG-mediated signaling can be positively regulated by ERRα in prostate cancer cells independent of their AR expression status.

### ERRα can directly transactivate T:E fusion gene in AR-positive and AR-negative prostate cancer cells

**Fig. 5:**
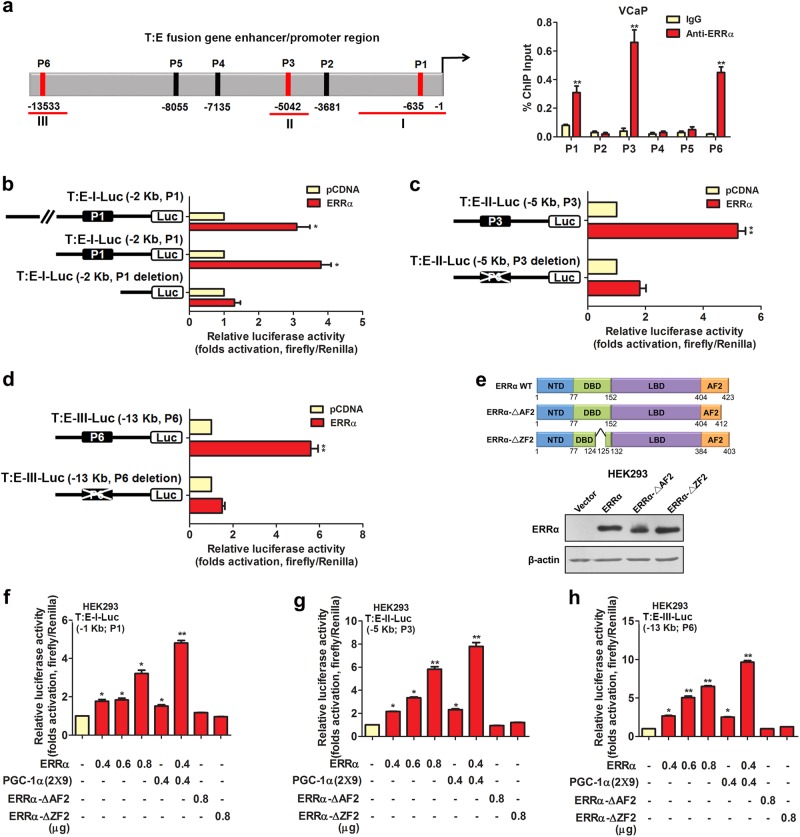
ERRα can directly transactivate the T:E fusion gene in AR-positive prostate cancer cells. **a** ChIP assay of T:E fusion gene performed in AR-positive VCaP cells. Left panel: schematic diagram shows the predicted ERRα-binding sites (P1–P6) located in the promoter and enhancer regions of T:E fusion gene. Right panel: results validated that three sites, namely P1, P3, and P6, located respectively at −640 bp, −5 kb and −13.5 kb upstream of the T:E fusion gene transcription start site, were enriched of ERRα. **b**–**d** Luciferase reporter assays of T:E fusion gene regulatory regions performed in ERRα-transfected HEK293 cells. All three T:E-I/II/III-Luc reporter constructs, driven by different lengths of T:E promoter/enhancer fragments, could be significantly transactivated by ERRα. Deletion of ERRα binding motifs in the T:E-Luc reporters abolished or significantly reduced the ERRα-induced transactivation. **e** Upper panel: Schematic diagram shows the domain structures of the wild-type ERRα protein and its truncated mutants. Lower panel: Immunoblot detection of transfected ERRα and its truncated mutants in HEK293 cells. (**f**–**h**) Luciferase reporter assays of T:E-I/II/III-Luc constructs performed in HEK293 cells. All three reporter constructs could be dose-dependently transactivated by ERRα, with further potentiation by co-transfection with PGC-1α (2 × 9), but not by ERRα-ΔAF2 and ERRα-ΔZF2 truncated mutant constructs. ^*^P < 0.05; ^**^P < 0.01 versus empty vector pcDNA3.1; two-tailed Student’s *t* test. All data are presented as mean ± SEM obtained from three independent experiments

We next sought to determine whether up-regulation of T:E fusion gene or T:E-expressed ERG as induced by ERRα in prostate cancer cells could be mediated by direct transactivation of its gene or indirect regulation via other ERRα-regulated downstream signaling pathways. Sequence analysis predicted a total of six potential ERRα-binding sites in the promoter and enhancer region of T:E fusion gene, including four putative ERRα-binding element/ERRE-containing (P2–5 sites distributing over the region −3 to −8 kb upstream of *TMPRSS2* transcription start site) and two androgen-responsive element/ARE-containing sites (P1 and P6 sites located at −600 bp and −13 kb upstream of *TMPRSS2* transcription start site). ChIP assays performed in AR-positive VCaP cells validated that among these potential binding sites, ERRα could directly bind to three sites, namely the ERRE-containing P3 site and two ARE-containing P1 and P6 sites (Fig. 5a). ChIP assay performed in another T:E-positive but AR-negative NCI-H660 cells showed that ERRα could directly bind to the P3 site (Supplementary Fig. S6a). Previous studies show that besides ERRE-binding motifs, ERRα can bind to the ARE sites present in the AR-target *PSA* promoter [32]; and also the designated P1 and P6 binding sites as identified at the *TMPRSS2* gene promoter are characterized as the androgen-regulated motifs [33]. Based on our ChIP results, we generated three luciferase reporter constructs (T:E-I/II/III-Luc), which contained the T:E promoter or enhancer regions with the ERRE/ARE-containing sites (P1, P3, or P6), for reporter gene analysis. Reporter assays performed in HEK293 cells showed that these T:E-Luc reporter constructs, driven by different T:E promoter/enhancer fragments, could be significantly transactivated by the transfected ERRα (Fig. 5b–d), and their ERRα-induced transactivation could be inhibited by XCT790 (Supplementary Fig. S6b–d). However, deletion of the ERRE/AREs in these reporters abolished their transactivation, suggesting these binding motifs present at the T:E promoter/enhancer were essential for the ERRα-mediated transactivation. Further truncation analysis of the functional domains of ERRα showed that the T:E-Luc reporters could only be transactivated by the intact ERRα but not its AF2/DBD-truncated mutants, with further enhancement by cotransfection with ERRα-specific coregulator PGC-1α (2 × 9), suggesting that an intact ERRα would be required for the transactivation of T:E fusion gene (Fig. 5e–h). Additionally, transactivation of ARE-containing T:E-Luc-I/III-Luc reporter constructs could be further potentiated by cotransfection with AR (Supplementary Fig. S6e–g). To further address whether the ERRα binding to regulatory regions of T:E fusion gene would be affected by AR signaling, we next performed ChIP assay in VCaP cells with AR inhibition. Results showed that binding of ERRα to the T:E promoter-enhancer regions was not affected by AR knockdown or inhibition and conversely inhibition of ERRα by XCT790 did not affect AR binding to T:E regulatory regions (Supplementary Fig. S7a–c). These results suggest that the binding of ERRα and AR to the T:E fusion gene enhancer or promoter regions are independent of each other in prostate cancer cells. Finally, to elucidate whether the promoter–enhancer regions would interact mutually or form DNA looping in the ERRα-mediated transactivation of T:E fusion gene, we performed the ChIP-3C assay in VCaP cells. Results showed that a PCR product could be amplified in chromatin religated between promoter and enhancer (P6) regions of T:E fusion gene and the PCR signal was significantly suppressed in VCaP cells upon ERRα inhibition (XCT790) but not AR inhibition (siAR or enzalutamide) (Supplementary Fig. S7d), suggesting that ERRα could induce a promoter-enhancer DNA looping in transactivation of T:E fusion gene in prostate cancer cells. Together, these results suggest that ERRα can directly transactivate the T:E fusion gene via its direct binding to multiple binding motifs in the *TMPRSS2* promoter.

### Enhanced T:E fusion-mediated invasion and metastasis capacity in AR-negative prostatic cells is under ERRα regulation

**Fig. 6:**
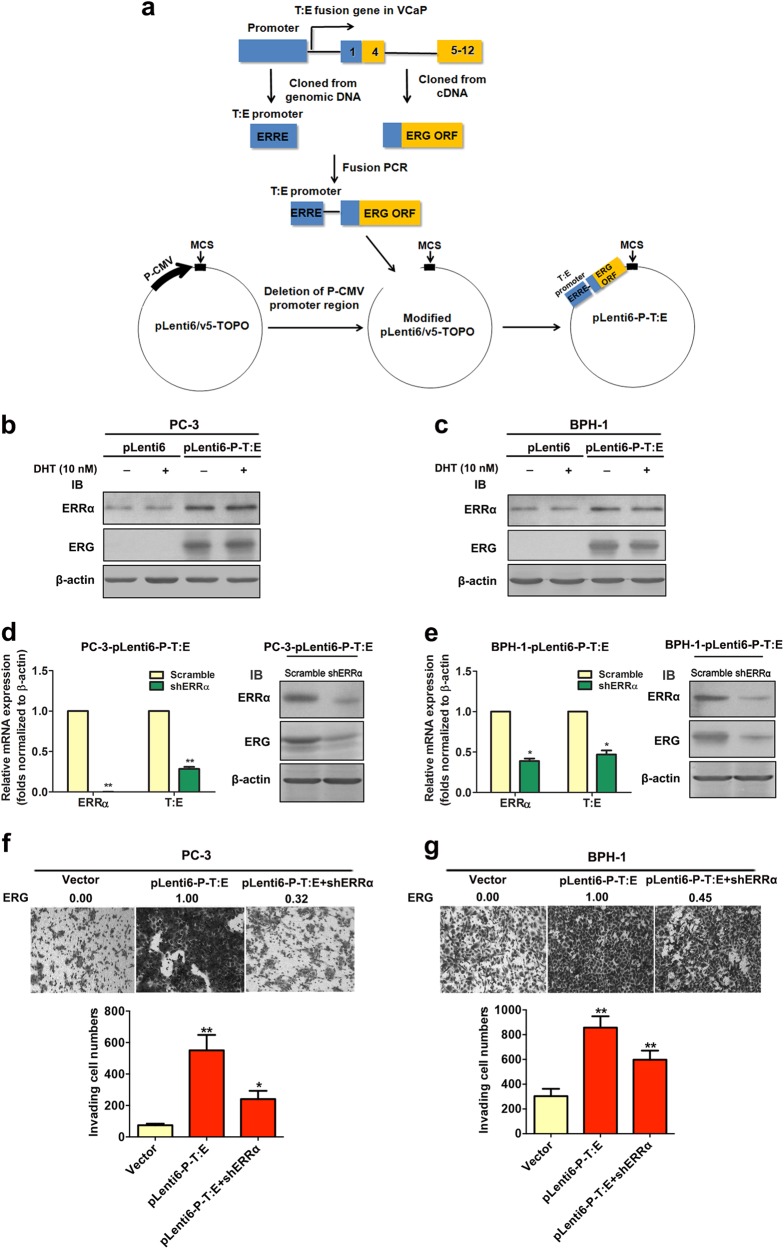
Enhanced invasion capacity mediated by ectopic T:E fusion or ERG in AR-negative prostatic cells is under ERRα regulation. **a** Schematic diagram shows the construction of the lentiviral plasmid expressing the T:E fusion or ERG under the regulation of ERRα. An approximate 2-kb fragment of the innate T:E fusion gene promoter including the P1 ERRα-binding element and the full-length open reading frame (ORF) of the T:E fusion gene was cloned from the genomic DNA and cDNA respectively from the T:E fusion-positive VCaP cells. The two DNA fragments were PCR-ligated and inserted into the modified CMV promoter-deleted lentiviral expression vector pLenti6-V5-TOPO as pLenti6-P-T:E. Under the regulation of its innate promoter containing the P1 ERRα-binding element, the generated pLenti6-P-T:E expresses the T:E fusion or ERG. **b** and **c** Immunoblot analyses of ERRα and ERG in T:E fusion stable infectants of PC-3 and BPH-1 cells. Results validated that ERG was expressed in both PC-3-T:E and BPH-1-T:E infectants, with no alteration upon dihydrotestosterone (DHT) treatment. **d** and **e** RT-qPCR and immunoblot analyses of ERRα and ERG in ERRα-knockdown T:E infectants. Results showed that both mRNA and protein levels of ERG were significantly reduced in both PC-3-T:E and BPH-1-T:E infectants upon shRNA-induced knockdown of ERRα. **f** and **g** Transwell invasion assay on PC-3-T:E and BPH-1-T:E infectants. Upper panels: representative images show the crystal violet-stained invading T:E infectants, magnification 100×. Analysis showed that both PC-3-T:E and BPH-1-T:E infectants exhibited significant higher invasion capacity than their control counterpart vector-infectants. The invasion capacities of both T:E infectants were significantly suppressed upon shRNA-induced knockdown of ERRα. Figures above the images indicate the relative expression levels of ERG in respective clones. ^*^P < 0.05; ^**^P < 0.01 versus empty vector-infectants, two-tailed Student’s *t* test. All data are presented as mean ± SEM obtained from three independent experiments

In order to elucidate the significance of ERRα in the regulation of T:E fusion gene and its contribution to the malignant growth capacity of prostate cancer cells without the AR influence, we generated stable T:E fusion-transduced infectants in three AR-negative prostatic cell lines (PC-3, BPH-1, and NCI-H660) using a T:E fusion expression plasmid pLenti-P-T:E, in which the ERG expression was driven specifically by a fragment of ERRα-regulating T:E promoter containing the P1 ERRα-binding site (Fig. 6a). Immunoblot analysis validated that only the PC-3-T:E and BPH-1-T:E infectants generated by the T:E promoter-containing pLenti-P-T:E plasmid positively expressed ERG, but not the promoter-less pLenti-T:E plasmid (Fig. 6b, c; Supplementary Fig. S8). Results also validated that the protein expression levels of ERG in these T:E fusion infectants were not affected upon DHT treatment, further demonstrating that the ectopic ERG expression in these T:E fusion infectants was solely regulated by ERRα but not AR. Furthermore, shRNA-mediated knockdown of endogenous ERRα resulted in a significant reduction of both mRNA and protein expression levels of ERG in these T:E infectants (Fig. 6d, e). Together, these results showed that the ectopic expression of ERG in these T:E infectants was under the regulation of ERRα but unresponsive to androgen. Since T:E fusion is associated with prostate cancer invasion [9], we next examined the effects of ERRα-regulated ectopic T:E fusion on the invasion capacity of prostatic cells. Transwell invasion assay showed that PC-3-T:E, BPH-1-T:E, and NCI-H600-T:E infectants exhibited higher invasion capacity as compared to their control vector-infectants and knockdown of ERRα resulted in reduced invasion capacity of T:E infectants (Fig. 6f, g; Supplementary Fig. S9). Our findings are consistent with a previous study that ectopic ERG overexpression can increase invasion capacity in benign immortalized (RWPE) and primary cultured (PrEC) prostatic epithelial cells [34]. In addition, in vivo tumorigenicity and metastasis studies on a highly metastatic AR-negative PC-3 subline PC-3M showed that ectopic T:E fusion expression in PC-3M cells could significantly promote their in vivo tumor growth and lymph node metastasis capacities in host SCID mice, as compared to their control counterpart PC-3M-vector/Luc^+^ infectants (Supplementary Fig. S10). Knockdown of endogenous ERRα in PC-3M-T:E-shERRα/Luc^+^ infectants could significantly suppress the T:E-induced in vivo malignant growth capacities. Together, our findings indicate that ectopic T:E fusion expression under the ERRα regulation can promote both the in vitro invasion and in vivo metastasis capacities of AR-negative prostatic cells and such T:E-mediated malignant growth potentials can be significantly attenuated by knockdown of endogenous ERRα.

### ERG can directly transactivate ERRα gene in prostatic cells

**Fig. 7:**
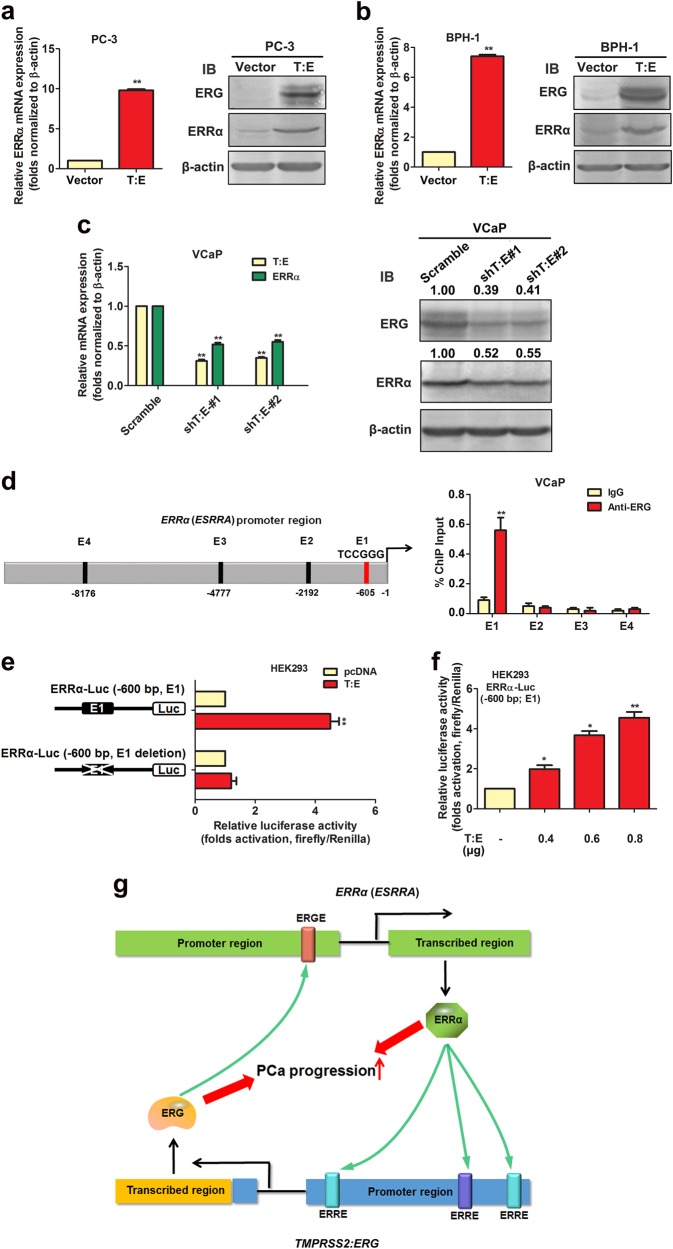
ERG can directly transactivate ERRα (*ESRRA*) gene in prostatic cells. **a** and **b** RT-qPCR and immunoblot analyses of ERG and ERRα expressions in T:E fusion infectants. Results showed that both mRNA and protein expressions of ERRα were significantly increased in both PC-3-T:E and BPH-1-T:E infectants. **c** RT-qPCR and immunoblot analyses of ERG and ERRα expressions in VCaP cells upon shRNA knockdown of T:E fusion. Results showed that both mRNA and protein expressions of ERRα were significantly reduced in VCaP cells upon shRNA knockdown of T:E. **d** ChIP assay. Left panel: schematic diagram shows the locations of potential ERG-binding sites (E1–E4), as predicted by promoter sequence analysis, at the promoter or regulatory region of ERRα gene. Right panel: ChIP assay performed in VCaP cells validated that only the E1 site but not others showed significant enrichment of ERG. **e** and **f** Luciferase reporter assays of ERRα gene promoter transactivation performed in T:E-transfected HEK293 cells. Results showed that the ERRα-Luc reporter, driven by a proximal ERRα gene promoter fragment containing the E1 element, could be significantly and dose-dependently transactivated by the transfected T:E. Deletion of the E1 element in the reporter abolished the T:E-induced transactivation of the reporter. ^*^P < 0.05; ^**^P < 0.01, two-tailed Student’s *t* test. All data are presented as mean ± SEM obtained from three independent experiments. **g** Schematic diagram shows the hypothesis on the reciprocal transactivations between ERRα and ERG expressed by the T:E fusion gene and their synergic contribution toward the prostate cancer progression

Intriguingly, we observed that T:E infectants of AR-negative prostatic cells expressed significant higher mRNA and protein expressions of ERRα as compared to their counterpart controls vector-infectants (Fig. 7a, b; Supplementary Fig. S9a). Furthermore, we also detected that shRNA knockdown of endogenous T:E fusion gene expression could decrease both mRNA and protein levels of ERRα in T:E fusion-positive VCaP and NCI-H660 cells (Fig. 7c, Supplementary Fig. S9b). Based on these findings, we hypothesize that the ERG expressed by T:E fusion gene could target to the ERRα gene in prostate cancer cells. Sequence analysis of the ERRα gene promoter predicted four potential ERG-binding sites (ERGREs, E1–E4), distributing over the 10-kb upstream of ERRα (*ESRRA*) promoter or regulatory region. To verify this, we performed the ChIP assays in the T:E fusion-positive VCaP cells. Results showed that among the four potential ERG-binding sites, the E1 site located at −635 bp upstream of the ERRα transcription start site was enriched of ERG (Fig. 7d). Furthermore, luciferase reporter assays performed in HEK293 cells, using a reporter construct ERRα-Luc driven by a 645-bp fragment of ERRα gene promoter (−645 to −1 bp) and a derived mutant construct with E1 deletion, showed that the ectopic transfected T:E fusion could induce the transactivation of ERRα-Luc reporter but not its E1-deleted mutant reporter (Fig. 7e). Reporter assay also showed that the ERRα-Luc (E1) reporter construct could be significantly and dose-dependently transactivated by the transfected T:E (Fig. 7f). These results manifested that the T:E fusion-expressed ERG could directly transactivate the ERRα gene in prostate cancer cells. Together with the direct ERRα targeting to the T:E fusion gene, our findings indicate that ERRα and ERG can form a reciprocal regulatory loop in their transactivations in prostate cancer cells and both cooperate with each other to advance the prostate cancer progression (Fig. 7g).

## Discussion

Although the expression of T:E fusion is closely associated with more aggressive tumors and higher rate of recurrence [7, 10, 11, 35–37], its clinical value as a reliable diagnostic and prognostic marker of prostate cancer still remains controversial [38–42]. Our present association results showed that overexpressions of both ERRα and ERG were closely associated with higher Gleason scores and metastasis in prostate cancer patients. It is apparent that increased expressions of both ERRα and ERG would contribute to a more aggressive phenotype of prostate cancer. Our data suggest that together with the demonstrated oncogenic roles performed by ERRα and ERG, combined evaluation of co-expressions of ERRα and ERG may provide a better prognosis for prostate cancer or predictor of sensitivity to hormone therapy.

Since the prostate cancer-prevalent T:E fusion gene is formed by the fusion with the androgen-responsive regulatory region of the AR-targeted *TMPRSS2* gene, it is generally viewed that T:E fusion is primarily regulated by AR and its aberrant expression in androgen-dependent prostate cancer is mainly attributed to the activated AR signaling, and AR targeting by androgen ablation or hormone therapy could help to suppress its aberrant overexpression in prostate cancer. However, its regulation wholly by AR in CRPC still remains elusive. In a previous study of patient-derived T:E-positive prostate cancer xenografts, Hermans et al. show that AR-positive and androgen-sensitive xenografts express high levels of T:E fusion transcripts, whereas some AR-negative and androgen-insensitive xenografts still express a basal level of ERG or no expression, and thus conclude that T:E fusion expression is functionally correlated with AR expression and its AR-dependent regulation may be bypassed in late-stage AR-negative prostate cancer [16]. In another study, Cai et al. [43] also show that ERG is expressed at comparable levels in T:E-positive androgen-dependent primary prostate cancer and androgen-independent CRPC; and in T:E-positive VCaP xenografts, its levels shows initial down-regulation in castrated mice but returns to precastration levels in relapse tumors. However, this proposed mechanism of action by AR activation cannot explain the expression of T:E fusion gene in advanced metastatic hormone therapy-resistant prostate cancer and AR-negative and androgen-independent prostate cancer cells [12, 16, 17, 21]. Moreover, in another study in clinical T:E-positive prostate cancer tissues, it is reported that prostatic T:E expression still remains persistent in some patients pre-treated with androgen ablation before surgery [44]. These contrasting results suggest a notion that T:E fusion expression may become androgen-independent or bypass in advanced prostate cancer and be regulated by other transcription factors or nuclear receptors. Indeed, our study of VCaP-CRPC xenograft model revealed that AR (full-length AR-FL and its spliced variant AR-V7), ERRα and ERG showed increased expressions in castration-relapse VCaP-CRPC tumors, with further increase of AR and ERRα upon enzalutamide treatment, but the AR signaling (shown by PSA level) did not show significant reactivation correspondingly (Supplementary Fig. S11). These results suggest that besides AR, ERRα may become a key regulator of T:E fusion gene in CRPC.

Here, we showed for the first time that ERRα can transactivate the T:E fusion gene via its direct binding to *TMPRSS2* promoter/enhancer in both AR-positive and -negative prostate cancer cells, with further potentiation by its coregulator PGC-1α. This direct regulation of T:E fusion by ERRα is independent of AR expression status in prostate cancer cells as evidenced by its transactivation by ERRα in AR-negative NCI-H660 cells. Since ERRα can cross-talk to AR signaling by its direct transactivation of common ARE-containing AR targets [32], it could be likely that the transactivation of T:E fusion gene by ERRα could be indirectly mediated through its interference on AR signaling in prostate cancer cells. Our data showed that suppression of ERRα by either XCT790 or shRNA-ERRα induced no change in AR mRNA levels in VCaP cells (ERα-negative and low ERβ) (Supplementary Fig. S4c, d), suggesting that the T:E suppression involves no perturbation of AR expression. Moreover, we also showed that ERRα inhibition by XCT790 could induce more or similar suppression of T:E expression as compared to AR inhibition or its knockdown in AR-positive VCaP cells, with or without stimulation with AR agonist (Supplementary Fig. S5). Together, our results suggest that the ERRα-mediated direct transactivation of T:E fusion gene would involve minimal or no cross-talk with AR in prostate cancer cells. Our present findings not only suggest a new role for ERRα in the regulation of T:E fusion expression in an AR-independent manner but also provide an insight on the persistent T:E expression in CRPC and androgen-independent prostate cancer [43, 44] in which AR-signaling is perturbed and ERRα shows an up-regulation pattern.

In a previous cDNA microarray expression profile study on two cohorts of prostate cancer patients, a fusion gene-specific signature is identified to be associated with ER signaling in T:E-positive prostate cancer tissues [22]. The authors also show by in vitro functional studies that the T:E fusion expression can be modulated by some ER ligands in T:E-positive NCI-H660 and VCaP cells, and hypothesize that the T:E expression can be down-regulated by an ERβ-dependent mechanism in prostate cancer cells. However, the significance of ER signaling in prostate cancer progression and the genuine value of ERα/β as prognostic markers still remains controversial. On the other hand, our present data showed that ectopic expression of either ERRα or its coactivator PGC-1α could induce significant increased expression of T:E but no change in ERα and ERβ in NCI-H660 cells (Supplementary Fig. S12), suggesting that the regulation of T:E fusion gene by ERRα was independent of ERs in prostate cancer cells, at least in AR-negative cells.

Intriguingly, our studies showed that ERRα and ERG could positively regulate each other at their gene promoters and appear to constitute a reciprocal loop in prostate cancer cells. This ERRα-ERG positive feedback loop between ERRα and ERG may help to explain their concomitant increased expression patterns in advanced prostate cancer. However, it still remains to further determine how this functional cooperation between ERRα and ERG could help to augment synergistically the signalings mediated by these transcriptional regulators respectively and also how the ERRα-ERG loop plays in the promotion and progression of prostate cancer, particularly therapy-resistance and metastasis.

Since T:E fusion gene is initially characterized as an androgen-regulated gene, hormone therapy against androgen signaling is considered as a therapeutic strategy for targeting the T:E-positive prostate cancer. However, expression study shows that T:E expression still remains persistent in clinical CRPC, CRPC xenograft tumors, and neuroendocrine prostate cancer [43]. Clinical association studies show no prediction on association between T:E fusion status and patient responsiveness to androgen-deprivation and antiandrogen therapy [45, 46]. Moreover, T:E expression status in circulating tumor cells isolated from metastatic CRPC patients shows no predictive response to androgen biosynthesis inhibitor abiraterone acetate [47]. All these studies manifest that hormone therapy against the androgen signaling is not enough to suppress or eradicate the T:E expression in CRPC. On the other hand, our present study suggests that targeting ERRα, which directly upstream regulates the promoter of T:E fusion, could be an attractive therapeutic approach to manage the T:E-positive prostate cancer.

In all, our present study suggests that the concomitant increased expressions of both ERRα and ERG, both are under a reciprocal transactivation feedback regulation between the two transcription factors, would perform a synergistic role in the advanced progression of prostate cancer (Fig. 7g). Our results also implicate that targeting ERRα could be a potential therapeutic strategy to suppress the T:E fusion gene expression in prostate cancer.

## Materials and methods

### Determination of clinical profiles of T:E fusion and ERRα in prostate cancer

The frequencies of T:E fusion and ERRα alterations in prostate cancer patients were analyzed using datasets from the Cancer Genome Atlas-Cancer Genomics and assessed by the cBioPortal for Cancer Genomics, which contain clinical genomic details of approximate 499 patients (TCGA, Provisional, TCGA, Cell 2015) [48]. The mutated TMPRSS2 contain ERG insert was defined ERG(+) in this study [49, 50].

### Cell lines and cell culture

Four human prostate cancer cell lines (LNCaP, VCaP, NCI-H660, and PC-3) and one immortalized prostatic epithelial cell line (BPH-1) were used in this study. 293FT and HEK293, and viral packaging cells PA317 were used in transfection and retroviral packaging, respectively. The culture media and conditions were followed as described previously [25, 51].

### Plasmid construction

(a) Expression plasmids: an approximate 2000-bp fragment of *TMPRSS2* gene promoter containing an ERRα-binding element (ERRE, P6) was fused with the full-length coding-domain sequence of *ERG* by PCR-fusion method as the T:E fusion and cloned into a CMV promoter-deleted pLentiV6 vector as pLenti-P-T:E for lentiviral transduction. pLenti-T:E without *TMPRSS2* gene promoter was also generated by insertion of *ERG* cDNA only into CMV-minus pLentiV6 vector. Expression plasmids of ERRα (pcDNA3-ERRα/ERRα-ΔZF2/ERRα-AF2) and PGC-1α (pcDNA3-PGC-1α/PGC-1α2 × 9) were constructed as described previously [27]. (b) shRNA plasmids: pLKO-shT:E#1/2 plasmids were cloned by inserting the synthetic oligonucleotide primers against T:E fusion into lentiviral vector pLKO.1; previously validated pLKO-shERRα plasmids were used for RNA interference [27]. (c) Reporter plasmids: human T:E gene promoter of lengths varying from −13 kb to −300 bp and ERRα (*ESRRA*) gene promoter (−987 to −1 bp) were PCR-amplified from genomic DNA of VCaP cells and cloned into pGL3 basic vector as pGL3-T:E-Luc and pGL3-ERRα-Luc for reporter gene assay.

### Lentiviral transduction

Procedures on generation of stable T:E fusion- and empty vector-transduced cells, and shT:E- and shERRα-transduced cells were followed as described previously [51, 52].

### Transwell invasion assay

Assay was performed on T:E- and shERRα-transduced prostatic cells following procedures as described previously [53, 54].

### RNA and protein analyses

Procedures on PCR and immunoblotting are followed as described previously [52, 55], and sequences of primer pairs for different target genes are listed in Supplementary Table S1. Primary antibodies used include ERG (abcam), ERRα (Epitomics), and β-actin (Santa Cruz).

### Molecular biology analyses

(a) Chromatin immunoprecipitation assay (ChIP). ChIP assays of endogenous T:E fusion gene and ERRα gene promoter were performed in VCaP cells following procedures as described previously [56]. Specific primer pairs for different target gene promoters are listed in Table S1. (b) ChIP-combined chromosome conformation capture assay (ChIP-3C). *Eco*RI was selected for enzyme digestion of chromatin after analysis of digestion map of the −14 kb enhancer/promoter region of T:E fusion gene. ChIP-3C assay of T:E fusion gene was performed in VCaP cells following a procedure as described previously [57]. Briefly, cross-linked chromatin was extracted from VCaP cells, sonicated, digested with *Eco*RI (TaKaRa) overnight and immunoprecipitated with protein A–agarose beads coupled with ERRα-antibody. The beads were precipitated, resuspended in ligation buffer and ligated with T4 DNA ligase (TaKaRa) overnight. The religated protein–DNA complexes bounded to beads were eluted, purified for DNA and analysis by PCR. (b) Luciferase reporter assay. Dual-luciferase assay was performed in HEK293 cells. After 48-h posttransfection, cells were further treated with XCT790 or without for 24 h, followed by measurement of luciferase activity as described previously [56].

### CRPC xenograft tumors

CRPC xenograft model VCaP-CRPC was established based on the castration-refractory growth of tumors formed by subcutaneous implantation of VCaP cells in castrated SCID mice as described previously [58].

### In vivo tumorigenicity and metastasis assays

In vivo tumorigenicity and metastasis assays were performed on empty vector-, T:E- or T:E-shERRα-transduced and firefly luciferase-labeled PC-3M-Luc^+^ cells following procedures as described previously [54].

### Statistical analysis

Student’s *t* test was used to compare the means between two groups. One-way ANOVA test was used to compare multiple groups. *P* < 0.05 was considered statistically significant.

## Electronic supplementary material

Supplementary Figures. S1–S12

Supplementary Figure Legends S1–S12

Supplementary Table S1
