# Supplementary material for: Nuclear receptor ERRα and transcription factor ERG form a reciprocal loop in the regulation of TMPRSS2:ERG fusion gene in prostate cancer
Source: Oncogene. 2018 Jul 24;37(48):6259–74. doi: 10.1038/s41388-018-0409-7 (PMC6265259; doi:10.1038/s41388-018-0409-7)
Supplement: Supplementary file 1 — Supplementary Figures. S1–S12 [file 41388_2018_409_MOESM1_ESM.pdf]

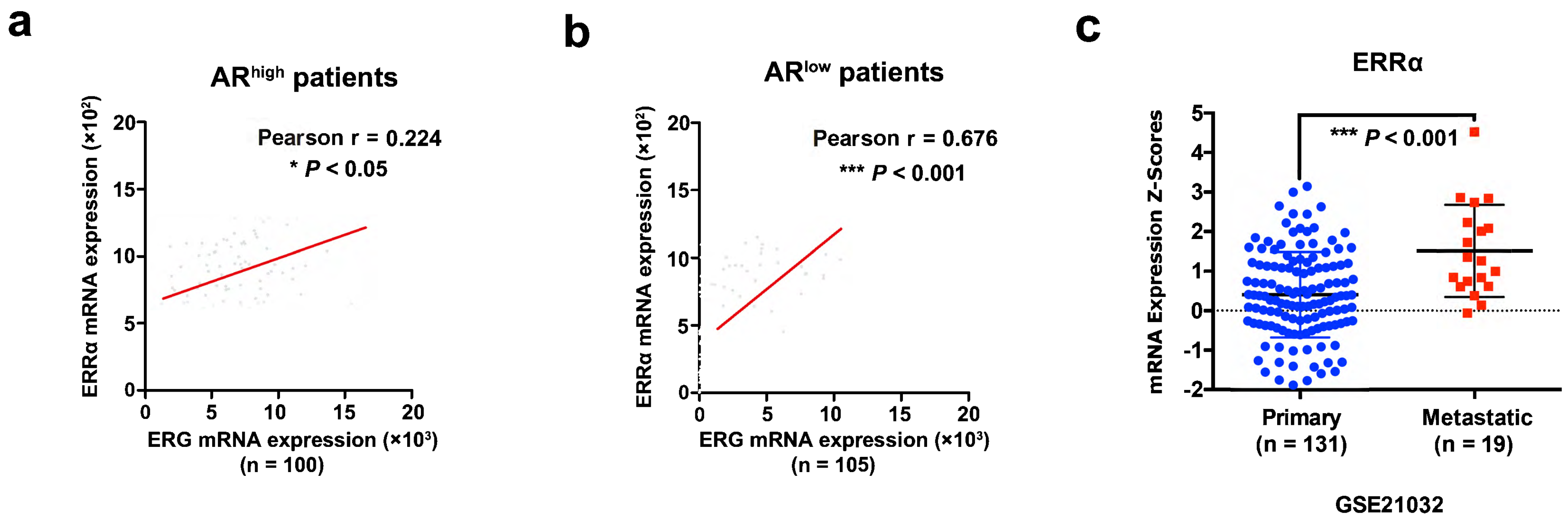

Supplementary Figure S1

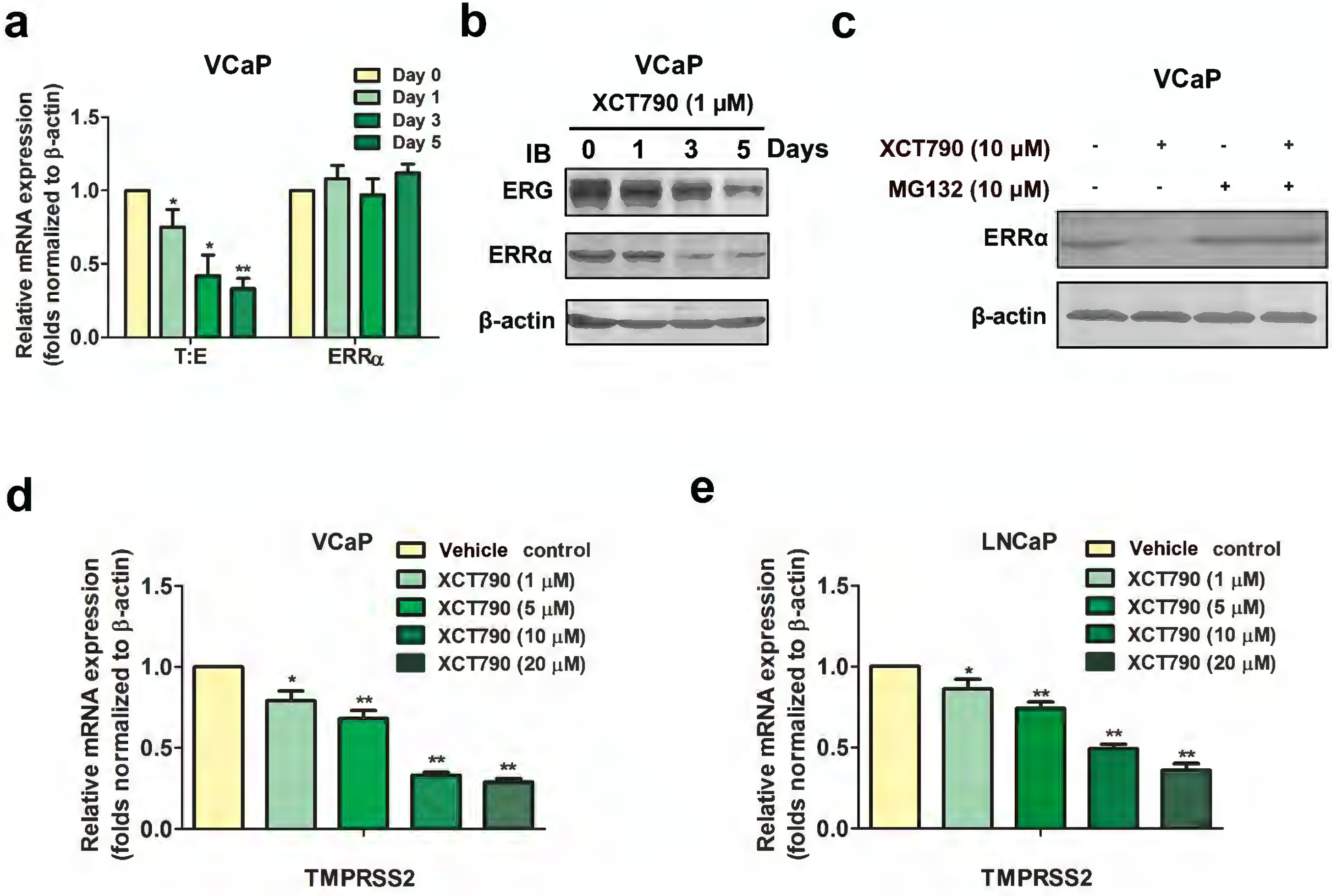

Supplementary Figure S2

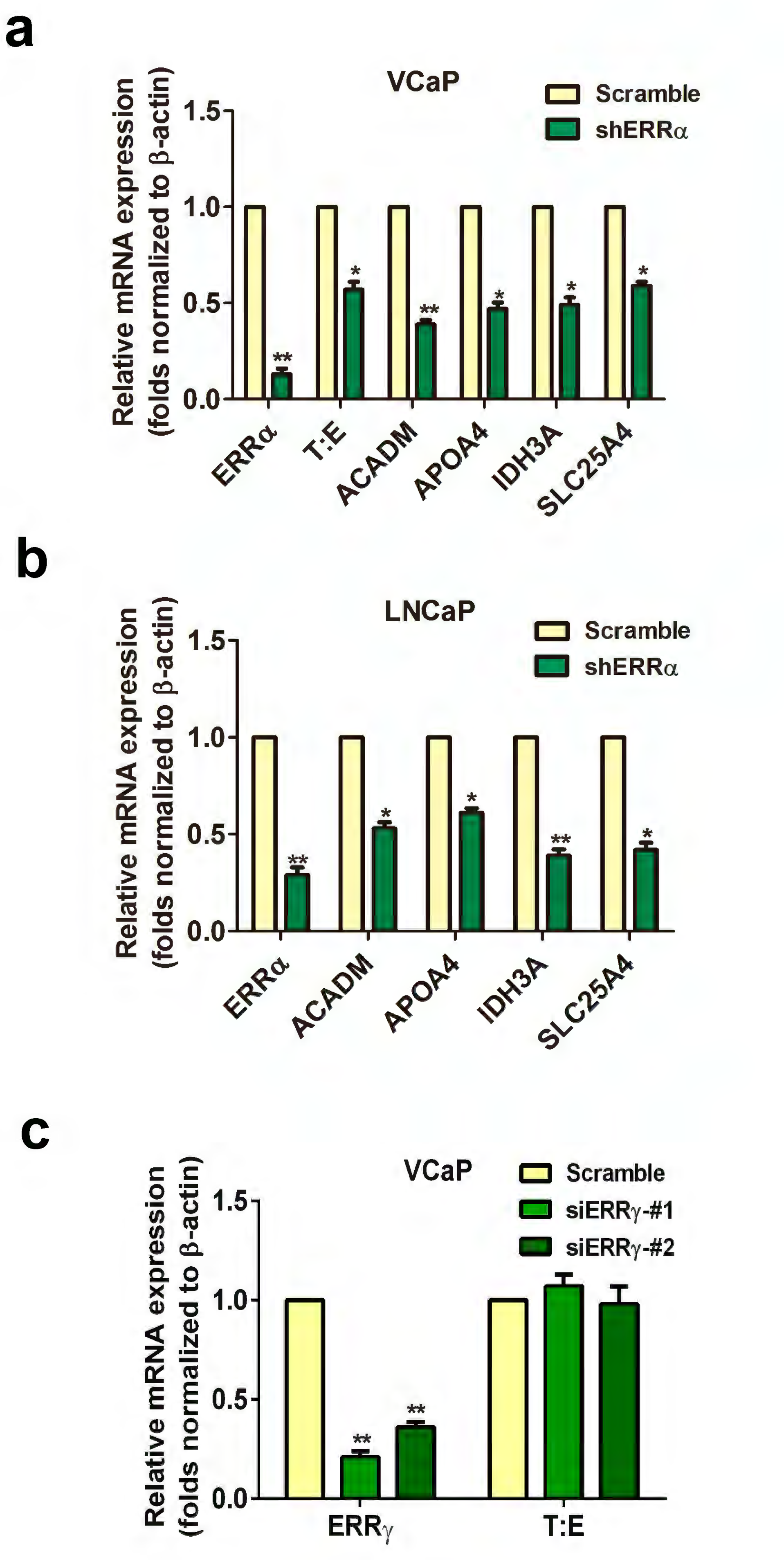

Supplementary Figure S3

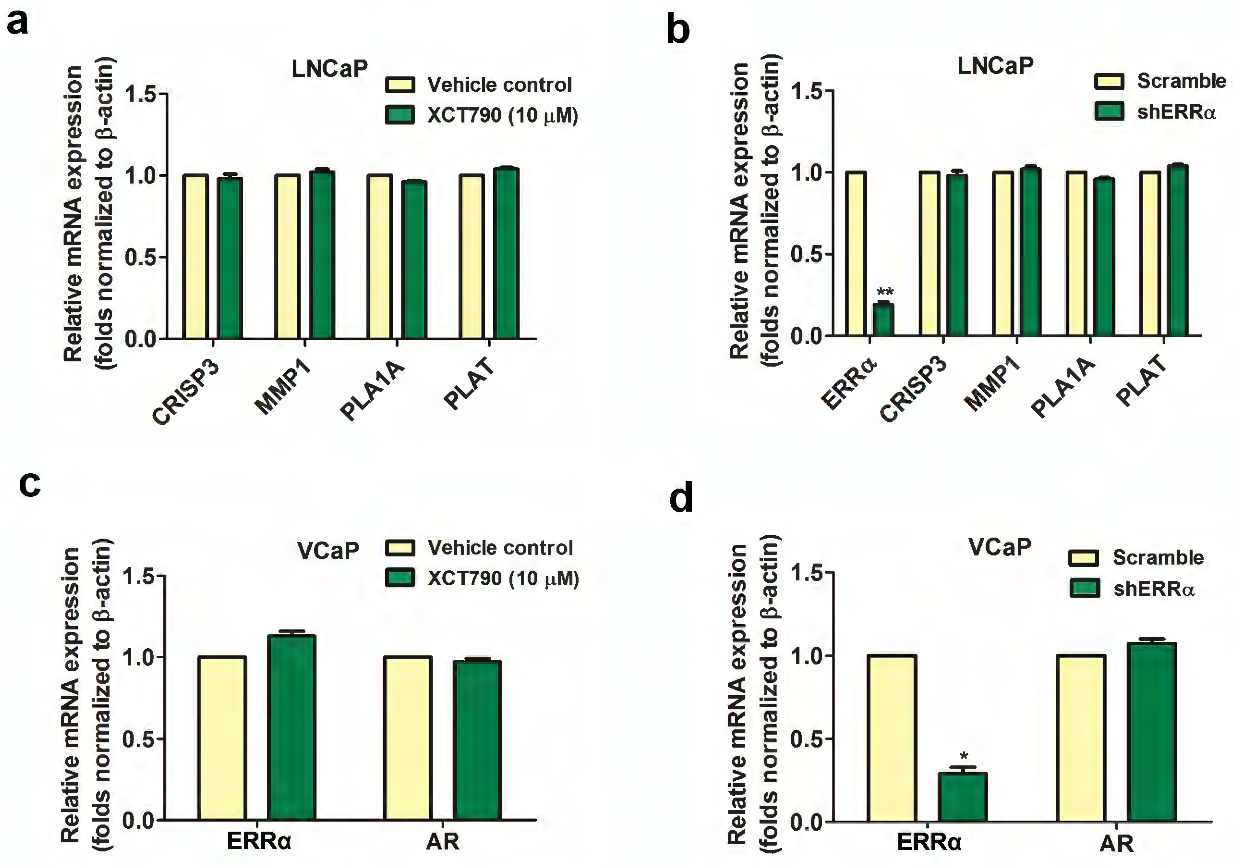

Supplementary Figure S4

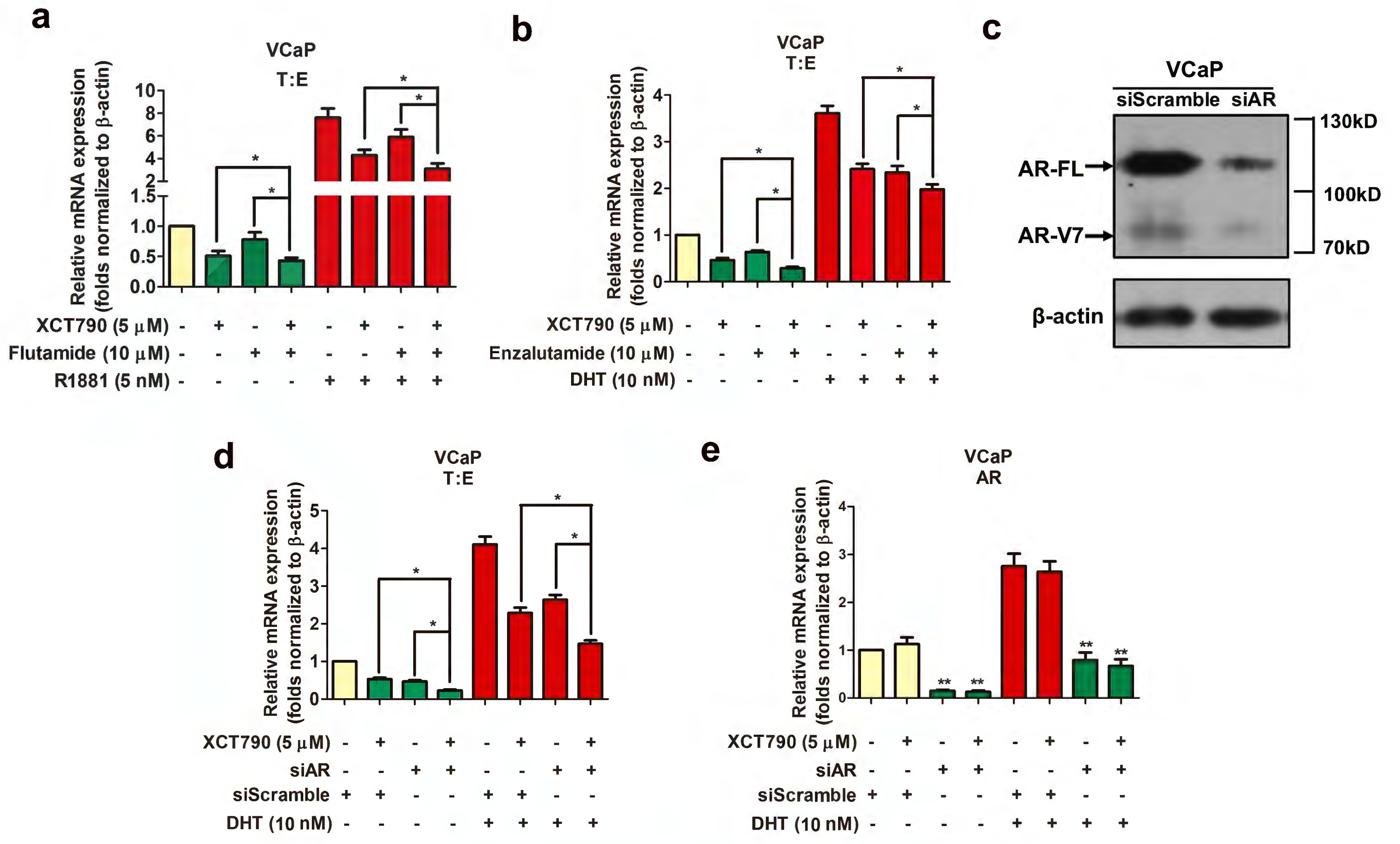

Supplementary Figure S5

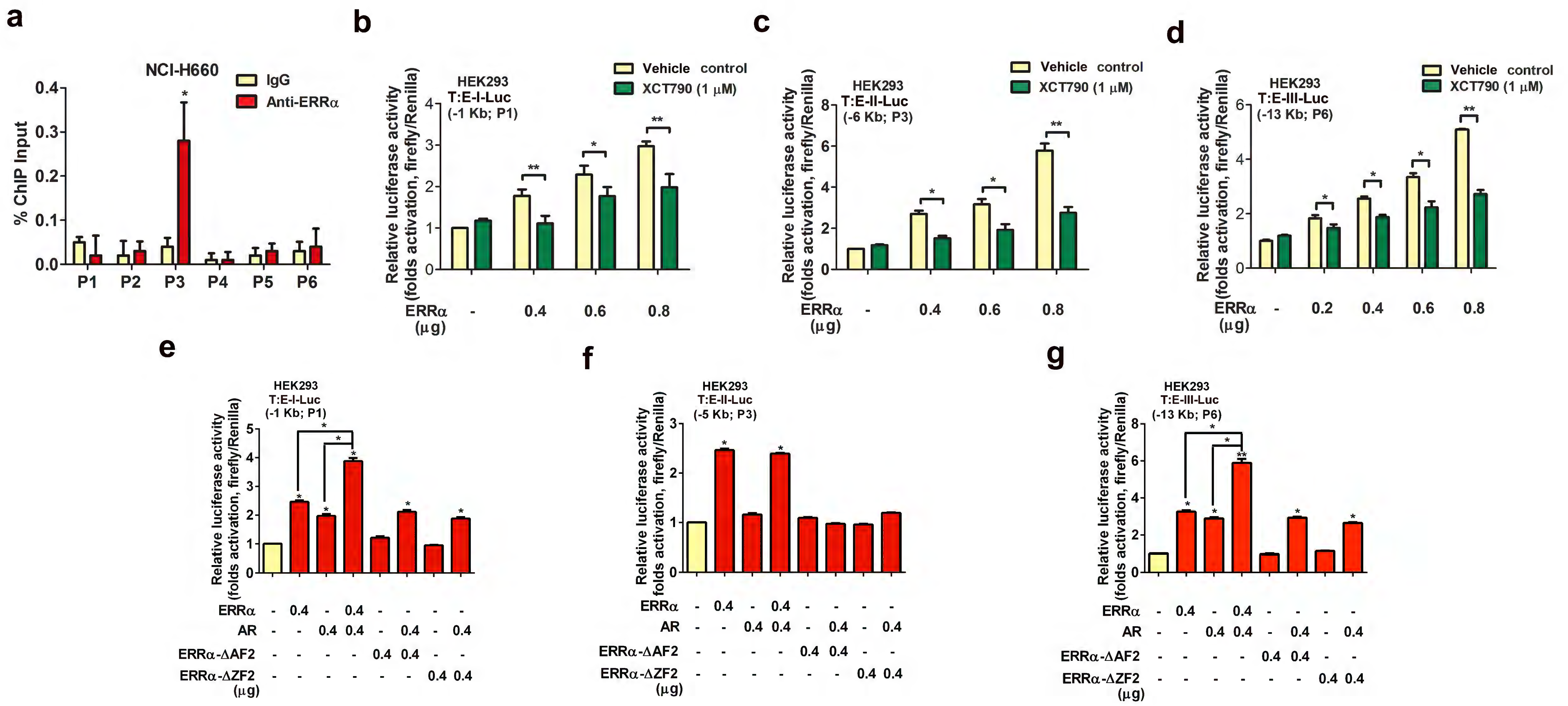

Supplementary Figure S6

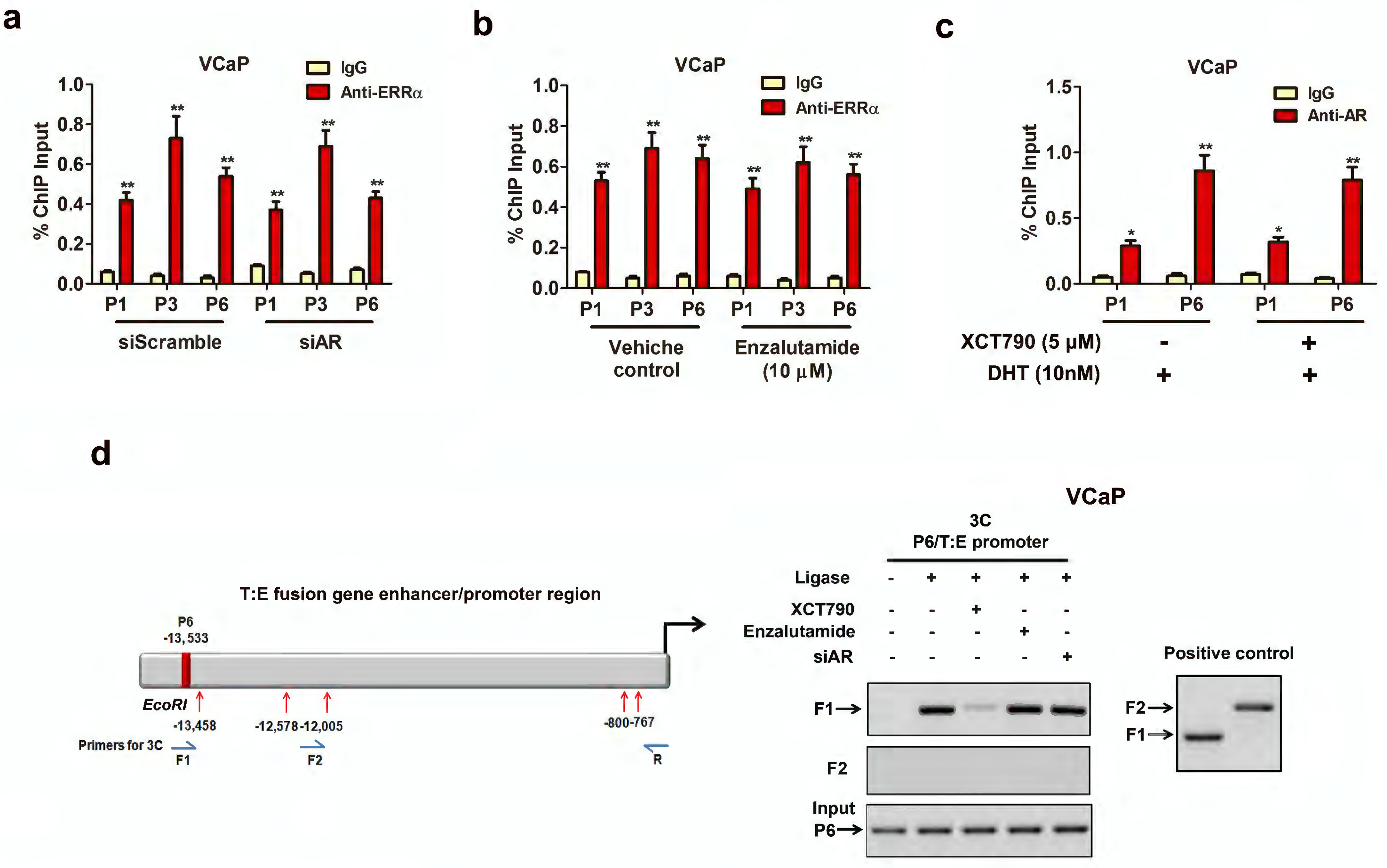

Supplementary Figure S7

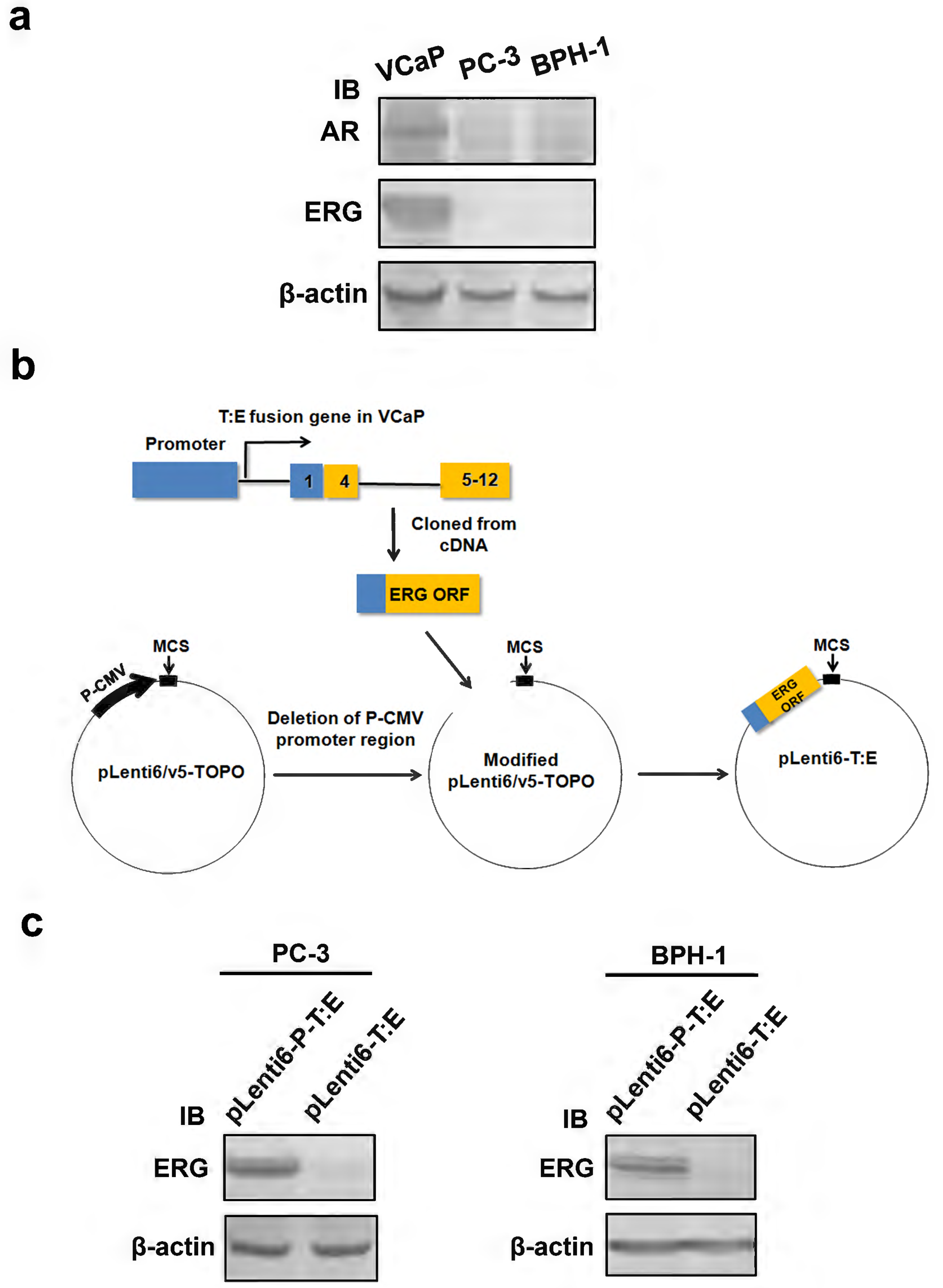

Supplementary Figure S8

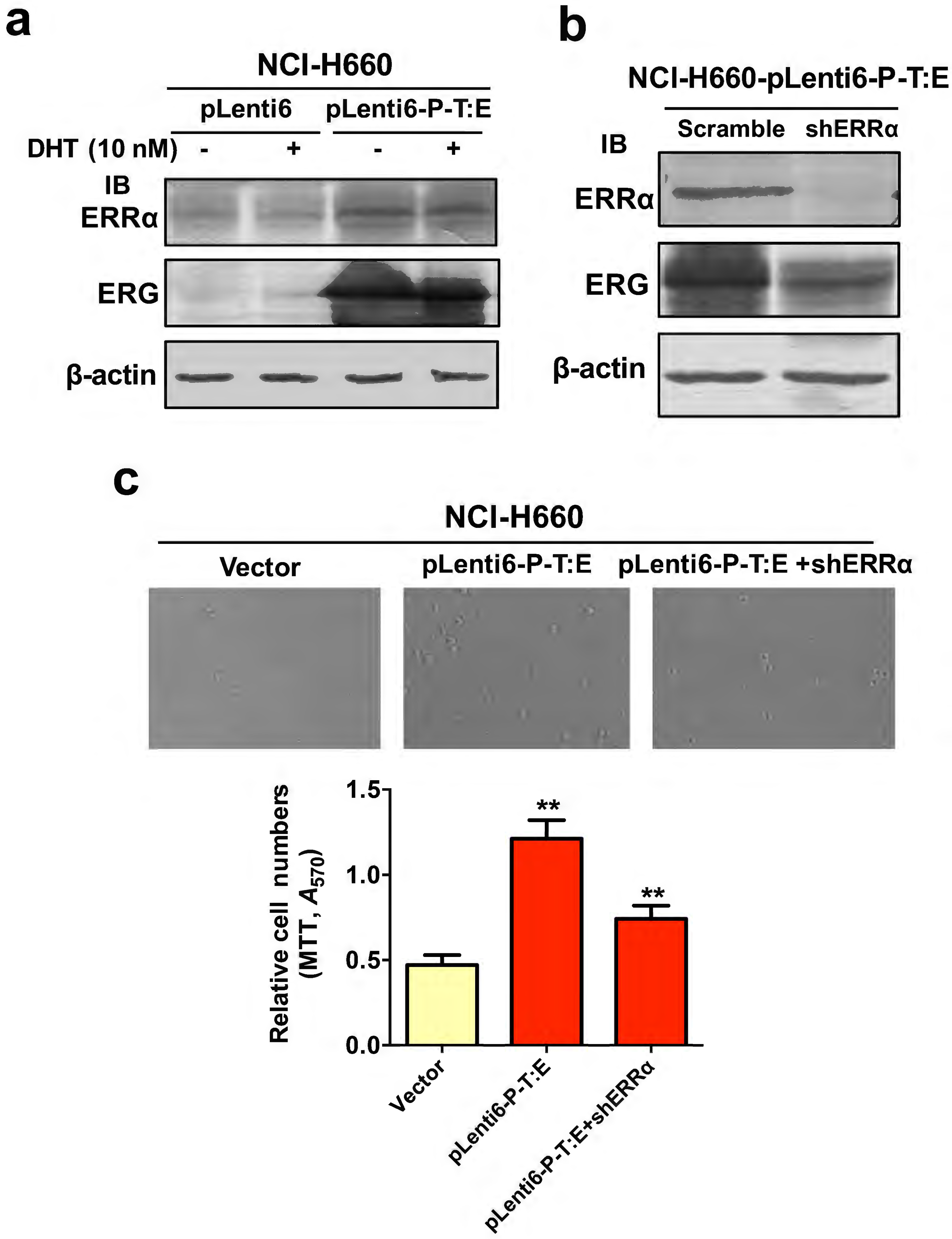

Supplementary Figure S9

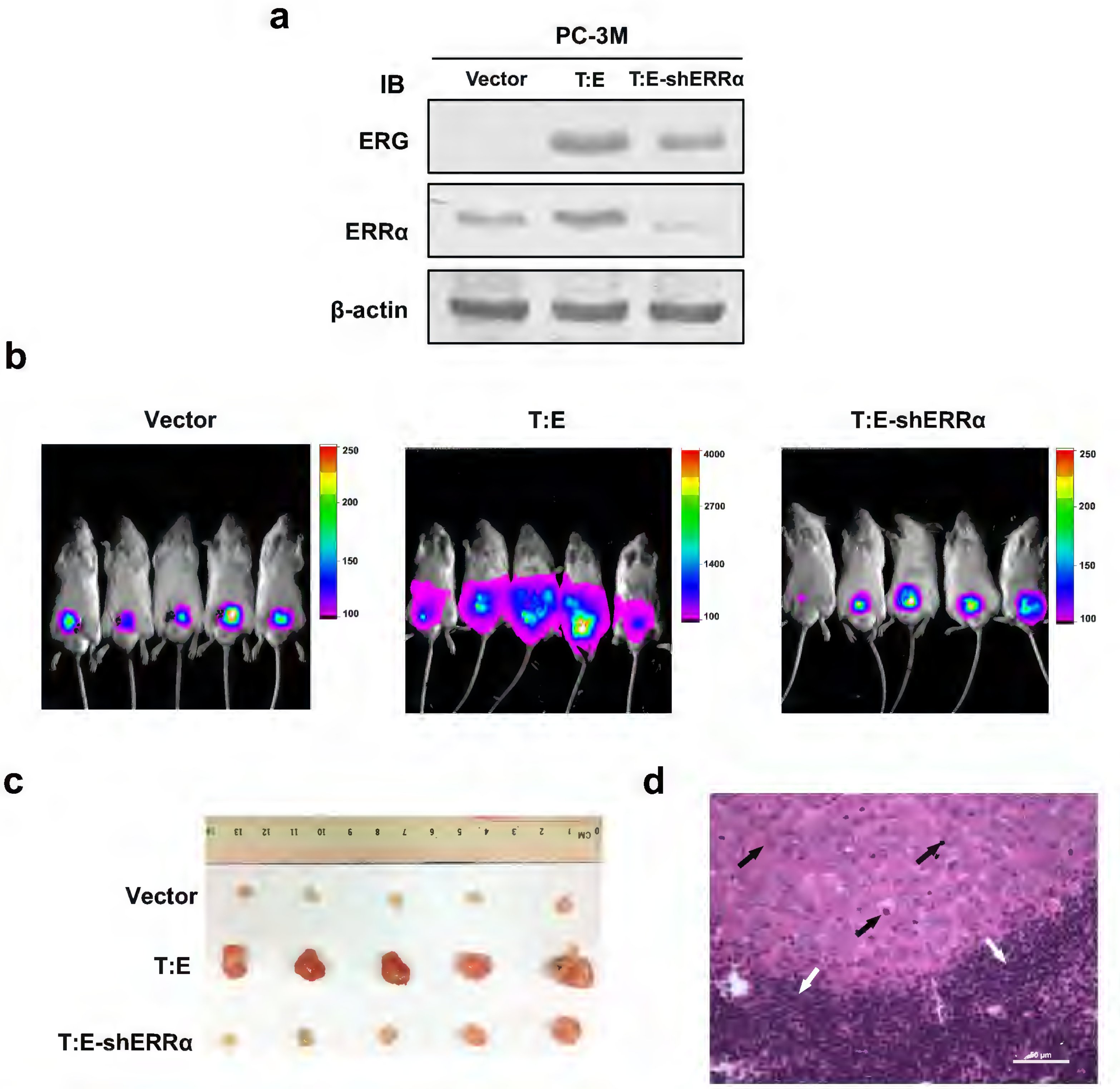

e

	PC-3M-vector n = 5	PC-3M-T:E n = 5	PC-3M-T:E-shERRα n = 5
Mean tumor weight (mg)**	48.3 ± 13.1	564.6 ± 154.3	170.5 ± 156.2
Lymph node metastasis (n) **	1/5	5/5	2/5

Supplementary Figure S10

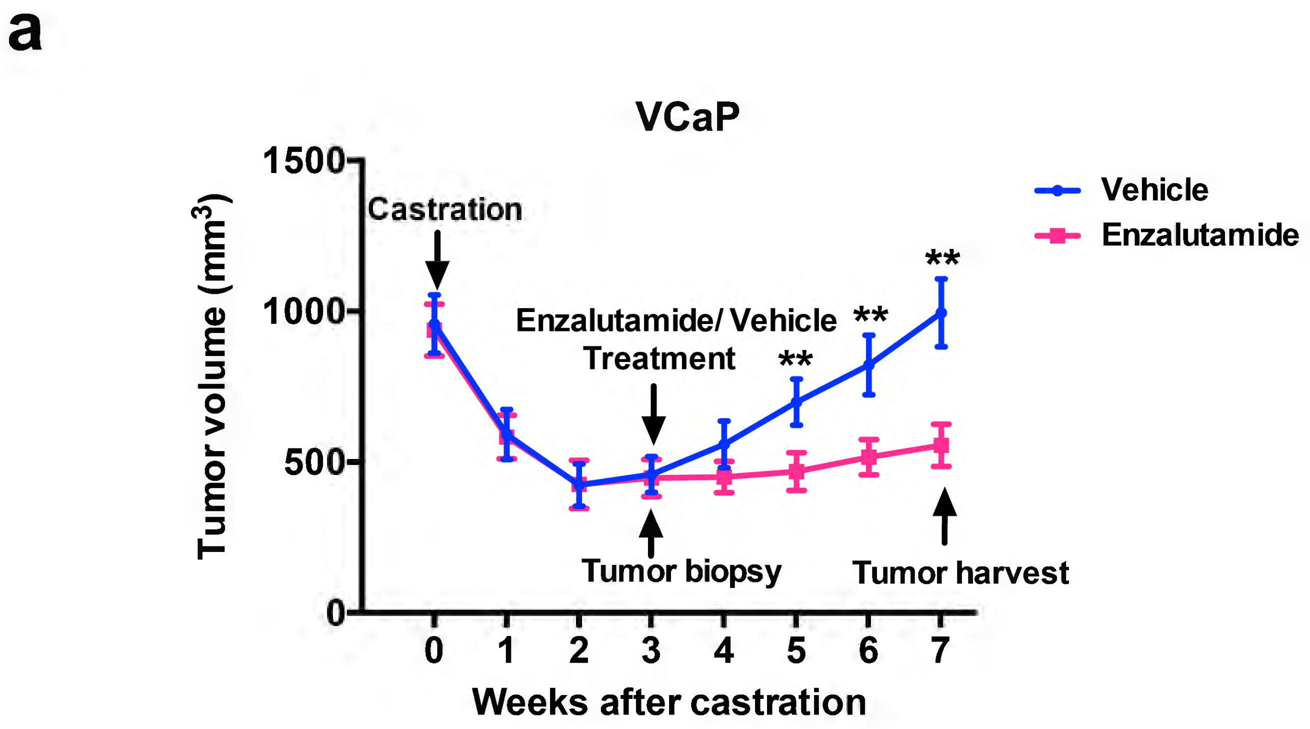

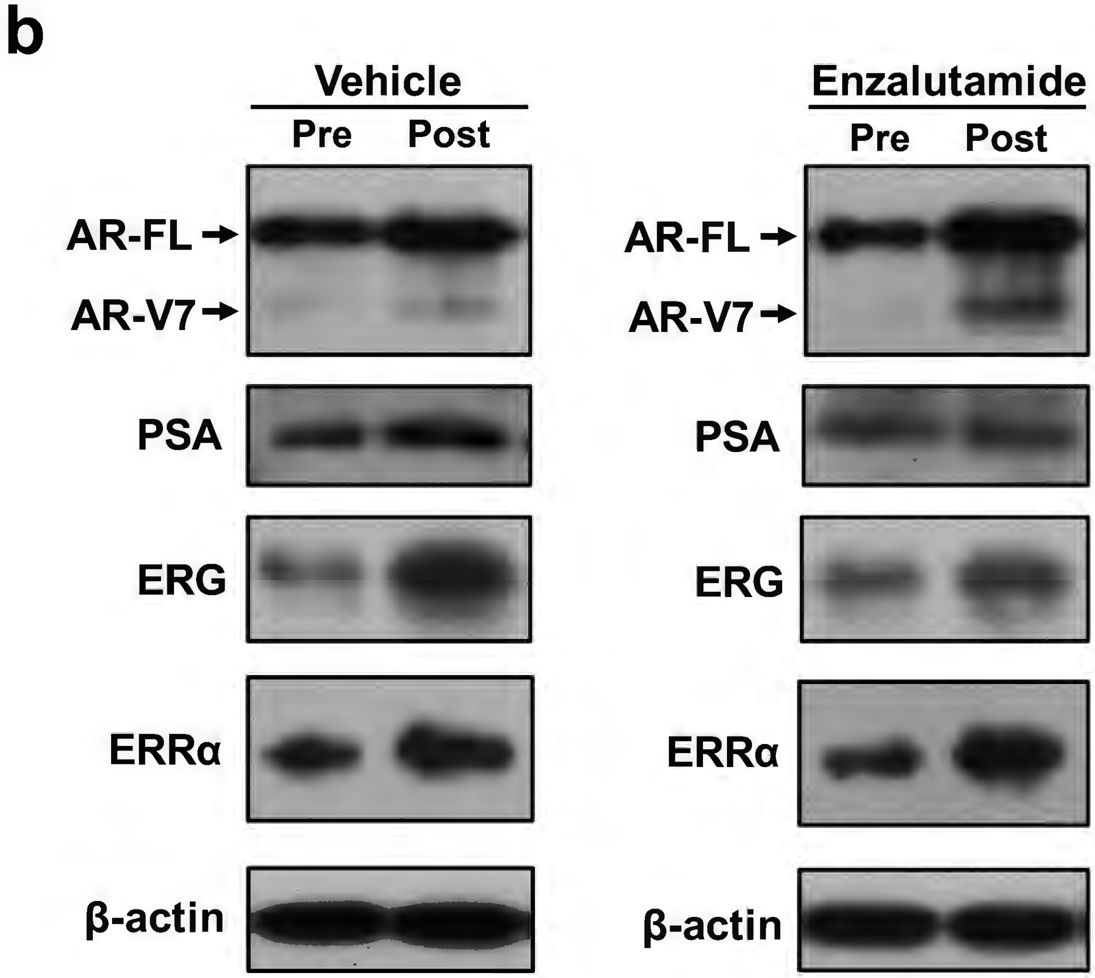

Supplementary Figure S11

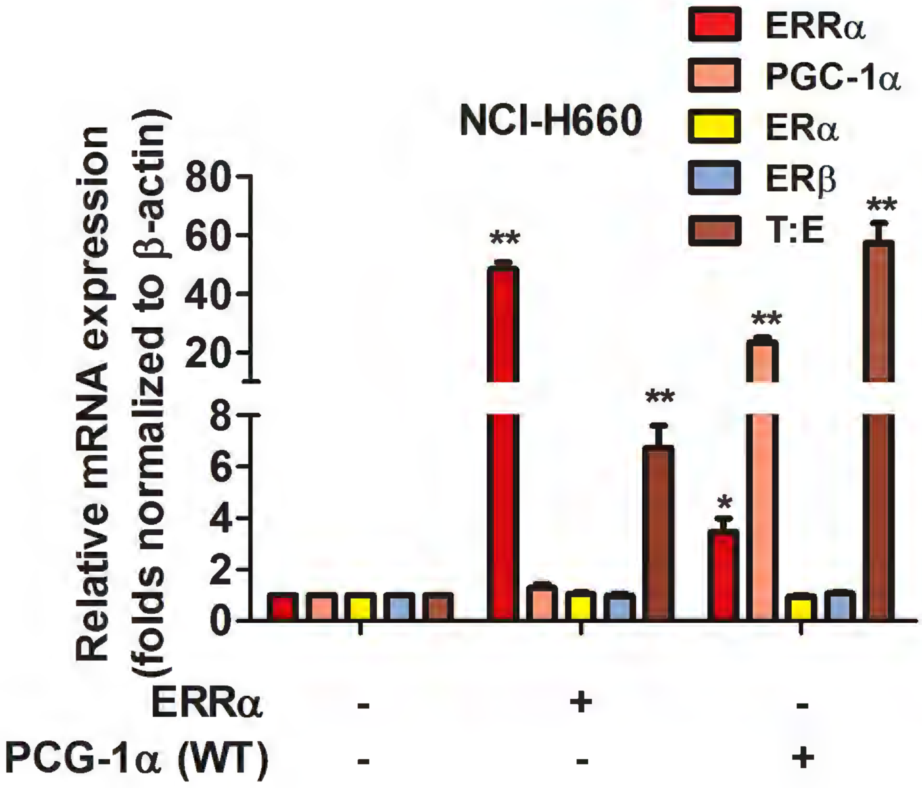

Supplementary Figure S12
