# Supplementary material for: Nuclear receptor ERRα and transcription factor ERG form a reciprocal loop in the regulation of TMPRSS2:ERG fusion gene in prostate cancer
Source: Oncogene. 2018 Jul 24;37(48):6259–74. doi: 10.1038/s41388-018-0409-7 (PMC6265259; doi:10.1038/s41388-018-0409-7)
Supplement: Supplementary file 2 — Supplementary Figure Legends S1–S12 [file 41388_2018_409_MOESM2_ESM.pdf]

Nuclear receptor ERRα and transcription factor ERG form a reciprocal loop in the regulation of *TMPRSS2:ERG* fusion gene in prostate cancer

Zhenyu Xu, Yuliang Wang, Zhan Gang Xiao, Chang Zou, Xian Zhang, Zhu Wang, Dinglan Wu, Shan Yu, and Franky Leung Chan

Supplementary Figure Legends

Supplementary Figure S1. Pearson correlation and linear regression analysis between ERRα and ERG expression in primary prostate cancer samples (TCGA, Provisional). Results showed that mRNA levels of both ERRα and ERG displayed a positive correlation in subsets of prostate cancer patients with either (**a**) AR^{low} or (**b**) AR^{high} expressions (high and low are defined by median value of AR mRNA level). (**c**) Expression profile of ERRα in primary and metastatic prostate cancer as revealed from a GEO dataset (GSE21032). Results showed that the ERRα mRNA level exhibited a significant increase in metastatic prostate cancer tissues as compared to primary tumors . *, $P < 0.05$; ***, $P < 0.001$.

Supplementary Figure S2. Treatment with XCT790 induces reduction of protein levels of both ERG and ERRα in prostate cancer cells. (**a**) RT-qPCR and (**b**) immunoblot analyses of T:E fusion or ERG and ERRα in VCaP cells upon treatment with low-dose XCT790 (1 µM) for a time course of 1-5 days. Result of RT-qPCR showed that XCT790 treatment could induce a time-dependent decrease of mRNA levels of T:E fusion but not ERRα in VCaP cells. However, immunoblot results revealed that protein levels of both ERG and ERRα were decreased in XCT790-treated VCaP cells in a time-dependent manner. (**c**) Treatments of VCaP cells with XCT790 and a proteasome inhibitor MG132. Immunoblot results showed that the decrease of ERRα protein expression in XCT790-treated VCaP cells could be prevented by MG132. (**d** and **e**) RT-qPCR analysis of *TMPRSS2* gene expression in AR-positive prostate cancer cells upon XCT790 treatment. Results showed that XCT790 could dose-dependently

suppress the endogenous *TMPRSS2* expression in T:E-positive VCaP and T:E-negative LNCaP cells. *, $P < 0.05$; **, $P < 0.01$ versus vehicle treatment.

Supplementary Figure S3. (**a** and **b**) Stable knockdown of ERRα induced significant suppressions of multiple ERRα target genes (*ACADM*, *APOA4*, *IDH3A* and *SLC25A4*) in both VCaP and LNCaP cells. (**c**) siRNA Knockdown of ERRγ induced no change in T:E fusion expression in VCaP cells as analyzed by RT-qPCR. *, $P < 0.05$; **, $P < 0.01$ versus sh/siScramble constructs.

Supplementary Figure S4. Inhibition of ERRα activity or its knockdown induces no change in expressions of ERG-responsive targets in T:E-negative LNCaP cells, and also induces no change in AR expression in T:E-positive VCaP cells. (**a** and **b**) RT-qPCR analysis showed that the mRNA expressions of ERG-responsive targets exhibited no changes in T:E-negative LNCaP cells upon XCT790 (10 μM) or shERRα treatment. (**c** and **d**) Similarly, XCT790 treatment and ERRα knockdown induced no change in AR levels in VCaP cells. Data are presented as mean ± SEM obtained from three independent experiments. *, $P < 0.05$; **, $P < 0.01$ versus vehicle treatment or Scramble shRNA, 2-tailed Student's *t* test.

Supplementary Figure S5. Suppression of T:E fusion gene expression in VCaP cells by XCT790 treatment, with further suppression by co-treatments with non-steroidal antiandrogen (flutamide or enzalutamide) or siRNA knockdown of AR as analyzed by RT-qPCR. (**a** and **b**) XCT790 and antiandrogen treatments. Results showed that single treatment with either XCT790 or antiandrogen (flutamide or enzalutamide) could suppress the T:E fusion expression in VCaP cells stimulated or unstimulated with AR agonist R1881 or DHT. Results also revealed that combined treatments with XCT790 and antiandrogen could synergistically suppress the T:E expression in R1881/DHT-treated and untreated VCaP cells. (**c**) Immunoblot validation of decreased AR (both AR-FL and AR-V7) levels in siAR-transfected VCaP cells. (**d**) XCT790 and siAR treatments. Similarly, suppression of T:E expression was detected in VCaP cells

treated singly with XCT790 or siAR and with further suppression by combined XCT790-siAR treatment. (**e**) AR expression. XCT790 treatment induced no change in AR expression in VCaP cells treated or untreated with DHT. *, $P < 0.05$ versus vehicle or siScramble.

Supplementary Figure S6. Suppression of ERRα-induced transactivation of T:E-I/II/III-Luc reporters by XCT790. (**a**) ChIP assay of T:E fusion gene performed in AR-negative NCI-H660 cells. Results showed that only the P3 site located at -5 kb upstream of the transcription start site of T:E fusion gene was enriched of ERRα. (**b-g**) Luciferase reporter assays of T:E fusion gene regulatory regions performed in ERRα-transfected HEK293 cells. **(b-d)** All three T:E-Luc-I/II/III-Luc reporter constructs, driven by different lengths of T:E promoter/enhancer fragments, could be dose-dependently transactivated by the transfected ERRα. But their transactivations were attenuated by XCT70. **(e-g)** ARE-containing T:E-Luc-I/III-Luc reporter constructs but not the ERRE-containing T:E-Luc-II could be further potentiated by co-transfection with AR. *, $P < 0.05$; **, $P < 0.01$ versus vehicle control.

Supplementary Figure S7. (**a**-**c**) ChIP assays of the regulatory regions of T:E fusion gene performed in AR-positive VCaP cells. **a** and **b**: results showed that the binding of ERRα to the three regulatory sites (P1, P3 and P6) at the promoter and enhancer regions of T:E fusion gene was not affected by AR knockdown (siAR) or its inhibition (enzalutamide). **c**: results also showed that inhibition of ERRα by XCT790 did not affect the binding of AR to ARE-containing P1 and P6 sites in the presence of DHT. (**d**) ChIP-3C assay of T:E fusion gene enhancer region performed in VCaP cells upon treatments with XCT790, enzalutamide or siAR. Left panel: schematic diagram shows the locations of the *Eco*RI cut sites (indicated by arrows) and the primers designed for ChIP-3C assay at the enhancer (-14kb – -12k upstream) and promoter regions of T:E fusion gene. F1, F2, designed enhancer primers at the enhancer region; R, designed anchor primer at the promoter region. Right panel: results showed that a PCR product was only amplified using the R anchor primer and the F1 enhancer primer but not the

F2 enhancer primer in the re-ligated chromatin and its amplification signal was significantly reduced in VCaP cells treated with XCT790 but unaffected by enzalutamide or siAR. Synthetized T:E promoter fragments (-1 bp–-1.4 kb; 100 µg) were used as positive control to validate the F1 and F2 primers.

Supplementary Figure S8. Generation of T:E fusion infectants in ERG-negative prostatic cells (PC-3 and BPH-1) by the promoter-less pLenti-T:E plasmid. (**a**) Immunoblots of AR and ERG. Parental VCaP prostate cancer cells showed positive AR and ERG expressions, whereas parental PC-3 prostate cancer cells and BPH-1 immortalized prostatic epithelial cells showed absence of AR and ERG expression. (**b**) Schematic diagram shows the construction of the promoter-less lentiviral plasmid pLenti6-T:E. The pLenti6-T:E was generated by insertion of only the cDNA of the full-length open reading frame (ORF) of the T:E fusion gene (i.e. ERG) without its promoter into the CMV promoter-deleted pLenti6-V5-TOPO as the pLenti6-T:E. (**c**) Immunoblot analysis of ERG. Only the PC-3 and BPH-1 infectants generated by the pLenti6-P-T:E containing the T:E promoter expressed ERG expression, but not by the T:E promoter-less pLenti6-T:E.

Supplementary Figure S9. (**a**) Generation of T:E infectants in AR-negative and T:E-positive NCI-H660 prostate cancer cells. The NCI-H660-T:E infectants expressed significant higher levels of ERRα and ERG than their empty vector-infectants, with no changes in their levels upon DHT stimulation. (**b**) shRNA Knockdown of ERRα induced decreased expression of ERG in NCI-H660-T:E infectants as shown by immunoblot analysis. (**c**) Transwell invasion assay on NCI-H660-T:E infectants. Upper panel: representative images of invading free-floating NCI-H660 infectants. Magnification, × 100. Lower panel: quantification of the relative cell numbers of invading cells by MTT assay. Results showed that the NCI-H660-T:E infectants exhibited higher invasion capacity than their empty vector and T:E-shERRα infectants. **, $P < 0.01$ versus empty vector or T:E-shERRα infectants.

Supplementary Figure S10. Ectopic T:E fusion expression promotes *in vivo* tumor growth and lymph node metastasis of PC-3M prostate cancer cells, whereas knockdown of endogenous ERRα suppresses their T:E-induced *in vivo* malignant growth capacities. (**a**) Immunoblot validation of ERG and ERRα expressions in T:E fusion stable infectants of PC-3M cells with or without shRNA knockdown of ERRα. Results validated that ERG was expressed in PC-3M-T:E infectants and its level was significantly reduced upon ERRα knockdown in PC-3M-T:E-shERRα infectants. (**b**) Bioluminescence *in vivo* imaging. Representative bioluminescent images of mice at 6th-week post-intraprostatic inoculation of PC-3M-vector/Luc$^+$, PC-3M-T:E/Luc$^+$ or PC-3M-T:E-shERRα/Luc$^+$ infectants. Significant enhanced tumor growth was detected in the mouse prostate receiving orthotopic inoculation of PC-3M-T:E/Luc$^+$ infectants, as revealed by their higher intense bioluminescent signals, as compared to mice receiving orthotopic inoculation of vector counterpart PC-3M-vector/Luc$^+$ infectants. Significant reduction of bioluminescent signals of prostate tumors was also detected in mice receiving inoculation of PC-3M-T:E-shERRα infectants with stable ERRα knockdown, as compared to mice receiving PC-3M-T:E/Luc$^+$ infectants. Scale bars show the color intensity map for the bioluminescent signals expressed as photon counts. (**c**) Photograph shows the dissected prostate tumors formed by the inoculated PC-3M-vector/Luc$^+$, PC-3M-T:E/Luc$^+$ or PC-3M-T:E-shERRα/Luc$^+$ infectants. PC-3M-T:E/Luc$^+$ infectants formed significant larger tumors in host mice than the PC-3M-vector/Luc$^+$ or PC-3M-T:E-shERRα infectants. (**d**) Representative H&E micrograph of aortic lymph node with prostate cancer metastasis in mice receiving inoculation of PC-3M-T:E/Luc$^+$ infectants. Black arrows indicate the metastasizing prostate cancer cells and white arrows indicate the lymphocytes in lymph node. Magnification, × 400; bar, 50 µm. (**e**) Table summarizes the results of prostate tumor weights and lymph node metastasis detected in mice inoculated with PC-3M-vector/Luc$^+$, PC-3M-T:E/Luc$^+$ or PC-3M-T:E-shERRα/Luc$^+$ infectants. **, $P < 0.01$ versus PC-3M-T:E/Luc$^+$ infectants.

Supplementary Figure S11. Enzalutamide treatment of castrated host mice bearing VCaP-CRPC xenograft tumors induces further expressions of AR and ERRα. (**a**) Growth curve of VCaP xenograft tumors in castrated host mice upon enzalutamide or vehicle treatment. Once the VCaP-CRPC tumors showed rebound growth at 2nd-3rd week post-castration, tumor bearing mice were randomly assigned to daily oral gavage with enzalutamide (10 mg/kg) or vehicle (1% carboxymethyl cellulose, 0.1% Tween-80 and 5% DMSO in distilled water) for additional 4 weeks (n = 5). Results showed that enzalutamide treatment could significantly retard the castration-relapse growth of VCaP-CRPC tumors as compared to vehicle. (**b**) Immunoblot analysis of AR (full-length AR-FL and spliced variant AR-V7), PSA, ERG and ERRα in VCaP-CRPC tumors upon enzalutamide or vehicle treatment. Results showed that protein levels of AR-FL, AR-V7, PSA, ERG and ERRα showed significant increases in VCaP-CRPC tumors at 4th-week post-treatment (Post) with vehicle as compared to that at pre-treatment (Pre). Enzalutamide treatment (Post) could further increase expressions of AR-FL, AR-V7 and ERRα and moderately increase ERG but attenuate PSA up-regulation in VCaP-CRPC tumors.

Supplementary Figure S12. ERRα overexpression induces no change in ERα and ERβ expressions in ERG-positive and AR-negative NCI-H660 cells. RT-qPCR analysis showed that transfection of co-regulator PGC-1α could significantly up-regulate the expressions of ERRα and T:E fusion but not ERα and ERβ in NCI-H660 cells, and ERRα transfection induced no changes in mRNA levels of PGC-1α, ERα and ERβ in NCI-H660 cells. *, $P < 0.05$; **, $P < 0.01$ versus empty vector control.
