# Supplementary material for: Nuclear receptor ERRα and transcription factor ERG form a reciprocal loop in the regulation of TMPRSS2:ERG fusion gene in prostate cancer
Source: Oncogene. 2018 Jul 24;37(48):6259–74. doi: 10.1038/s41388-018-0409-7 (PMC6265259; doi:10.1038/s41388-018-0409-7)
Supplement: Supplementary file 3 — Supplementary Table S1 [file 41388_2018_409_MOESM3_ESM.pdf]

Nuclear receptor ERRα and transcription factor ERG form a reciprocal loop in the regulation of *TMPRSS2:ERG* fusion gene in prostate cancer

Zhenyu Xu, Yuliang Wang, Zhan Gang Xiao, Chang Zou, Xian Zhang, Zhu Wang, Dinglan Wu, Shan Yu, and Franky Leung Chan

Supplementary Table S1 Information of primers

**Primer name**	**Nucleotide sequences**
ACTB (β-actin)	Forward ATGGATGATGATATCGCCGCG
	Reverse CTCCATGTCGTCCCAGTTGGT
TMPRSS2:ERG (T:E)	Forward TAGGCGCGAGCTAAGCAGGAG
	Reverse GTAGGCACACTCAAACAACGACTGG
CRISP3	Forward CACAATGAACTGAGGAGAGCAGTATC
	Reverse TGTCTGTAATTGCACTGGTTTGC
MMP1	Forward TTTCATTTCTGTTTTCTGGCCA
	Reverse CATCTCTGTCGGCAAATTCGT
PLAT	Forward CACTGGGCCTGGGCAAACATA
	Reverse CACGTCAGCCTGCGGTTCTTC
PLA1A	Forward CCACCCCACAATGCCAGATAAAC
	Reverse TCCCAATAATGGTAGTCCGGTCTTTT
ESRRA (ERRα)	Forward GTCCAAAGGGTTCCTCGGAG
	Reverse GGATGCCACACCATAGTGGTA

TMPRSS2	Forward CAGGAGTGTACGGGAATGTGATGGT
	Reverse GATTAGCCGTCTGCCCTCATTTGT
AR	Forward ATCTTGTCCACCGTGTGTCT
	Reverse AGCCCTAACTGCACTTCCAT
ACADM	Forward AGAACCTGGAGCAGGCTCTGAT
	Reverse AGAACCTGGAGCAGGCTCTGAT
APOA4	Forward CACGGTGATGTGGGACTACTTC
	Reverse CCAAGTTTGTCCTGGAAGAGGG
IDH3A	Forward TCGGTGTGACACCAAGTGGCAA
	Reverse TTCGCCATGTCCTTGCCTGCAA
SLC25A4	Forward GCTGCCTACTTCGGAGTCTATG
	Reverse TGCGACTGCCGTCACACTCTG
ChIP-T:E-P1	Forward TGGTCCTGGATGATAAAAAA
	Reverse CAGGGAGACATACGCCCCAC
ChIP-T:E-P2	Forward GAAACATGGATCAAAGGTG
	Reverse CCTCACATTTCAATCTTCCT
ChIP-T:E-P3	Forward GATGAAAGGAGTTTTAGCCC
	Reverse ATGGGGATTCTGCCAGCTGC
ChIP-T:E-P4	Forward TTAGTAGAGACGGGGTTTCA
	Reverse AGTGGCTCACACCTGTAATC
ChIP-T:E-P5	Forward GGACTACCAAGGAGAAGCTA
	Reverse ATTAATAGCCCTGCCTGGAT

ChIP-T:E-P6	Forward TTCTGAGCCCCCACAATTGC
	Reverse GTGGTGGGACACACCTCAGC
ChIP-ERRα-P1	Forward TGGGAGTGGGGATTGTTGGT
	Reverse ATAGCAGAACACGAACGTGG
ChIP-ERRα-P2	Forward GGCCGCAACATTAGCAAGAC
	Reverse ATTGATTTCCATGGAGCTGC
ChIP-ERRα-P3	Forward AAGCCCAGGGTTGTAACCTC
	Reverse CGCAGACTCCTGGGCCTTCC
ChIP-ERRα-P4	Forward TCGGTCACCGCATGGACCTT
	Reverse GCTGCGGAACTCGGGGTGGG
3C-R	AGGAAGACGTGGCTGTTCC
3C-F1	ACACAGCAAGGCAGAGGACA
3C-F2	CCAGTCTGTGGTATTCTGTA
pLKO-sh-T:E-#1	Forward CCGGTGACATCCTTCTCTCACATCTCG AGATGTGAGAGAAGGATGTCTTTTTG
	Reverse AATTCAAAAAGACATCCTTCTCTCACA TCTCGAGATGTGAGAGAAGGATGTCA
pLKO-sh-T:E-#2	Forward CCGGTGCCCATCAACAGACGTTGATAC TCGAGTATCAACGTCTGTTGATGGGCT TTTTG
	Reverse AATTCAAAAAGCCCATCAACAGACGT TGATACTCGAGTATCAACGTCTGTTGA TGGGCA
siAR	CAAGGGAGGTTACACCAAA

siERRγ-#1	AATGGCCATCAGAACGGACTT
siERRγ-#2	AACTTGTCTATGCAGACGATT
pGL3-T:E(-13kb,I)	Forward GCCTTGTGACACTTCACCCATCTTTGACA
	Reverse GCTGTTCTGCTCCCATTCTGTCCCCA
pGL3-T:E(-13kb,I)-deletion	Forward ACAGCAAGGCGCACTCTGTTGTGGGGCG
	Reverse CGCCCCACAACAGAGTGCGCCTTGCTGT
pGL3-T:E(-5000bp,II)	Forward CTGGATAGTCAGGGACTTGGAAGA
	Reverse TCCATGAGCCCATTGGGTGATGACAG
pGL3-T:E(-5000bp,II)-deletion	Forward CAGGCTGGTCTTGAACCTCCACCGACCT
	Reverse AGGTCGGTGGAGGTTCAAGACCAGCCTG
pGL3-T:E(-2000bp,III)	Forward CACTCCAAGAGGCCTGAGTC
	Reverse CGGCCCGCCCTGGCTCTCGCTTTA
pGL3-T:E(-1000bp, III)	Forward GGAGAGGAGGTGTCTGGTGA
	Reverse CGGCCCGCCCTGGCTCTCGCTTTA
pGL3-T:E(-300bp)	Forward ATCCCCTTCCTTAACCCAAA
	Reverse CGGCCCGCCCTGGCTCTCGCTTTA
pGL3-ERRα(-600bp)	Forward ATCGGCTGGGACCACTTCCTGACT
	Reverse CGCGCGAGAGGAGTGGGCGAGGCGGTG

pGL3-ERRα(-600bp)-deletion	Forward	CCTATCGGCCCTACTCGAATCCAGGAC
	Reverse	GTCCTGGATTCGAGTAGGGCCGATAGG
pcDNA3-T:E	Forward	CGCGAGCTAAGCAGGAGGC
	Reverse	CCTCCGCCAGGTCTTTAGTA
pLenti6-P-T:E-promoter	Forward	CACTCCAAGAGGCCTGAGTC
	Reverse	CGGCCCGCCCTGGCTCTCGCTTTA
pLenti6-P-T:E-cDNA	Forward	CGCGAGCTAAGCAGGAGGC
	Reverse	CCTCCGCCAGGTCTTTAGTA
pLenti6-P-T:E-fusion	Forward	AGGGCGGGCCGGGCCGGACTAGTCGCGAGCTAAGC
	Reverse	GCTTAGCTCGCGACTAGTCCGGCCCGGCCCGCCCT
